# Surface Water CO_2_ variability in the Gulf of Mexico (1996–2017)

**DOI:** 10.1038/s41598-020-68924-0

**Published:** 2020-07-23

**Authors:** Andrea K. Kealoha, Kathryn E. F. Shamberger, Steven F. DiMarco, Kristen M. Thyng, Robert D. Hetland, Derek P. Manzello, Niall C. Slowey, Ian C. Enochs

**Affiliations:** 1 Department of Oceanography, Texas A&M University, College Station, TX 77843 USA; 2 Department of Science, Technology, Engineering and Mathematics, University of Hawaii Maui College, Kahului, HI 96732 USA; 3 NOAA’s Atlantic Oceanographic and Meteorological Laboratory, Miami, FL 33149 USA

**Keywords:** Ocean sciences, Marine chemistry

## Abstract

Approximately 380,000 underway measurements of sea surface salinity, temperature, and carbon dioxide (CO_2_) in the Gulf of Mexico (GoM) were compiled from the Surface Ocean CO_2_ Atlas (SOCAT) to provide a comprehensive observational analysis of spatiotemporal CO_2_ dynamics from 1996 to 2017. An empirical orthogonal function (EOF) was used to derive the main drivers of spatial and temporal variability in the dataset. In open and coastal waters, drivers were identified as a biological component linked to riverine water, and temperature seasonality. Air-sea flux estimates indicate the GoM open (− 0.06 ± 0.45 mol C m^−2^ year^−1^) and coastal (− 0.03 ± 1.83 mol C m^−2^ year^−1^) ocean are approximately neutral in terms of an annual source or sink for atmospheric CO_2_. Surface water pCO_2_ in the northwest and southeast GoM open ocean is increasing (1.63 ± 0.63 µatm  year^−1^ and 1.70 ± 0.14 µatm year^−1^, respectively) at rates comparable to those measured at long-term ocean time-series stations. The average annual increase in coastal CO_2_ was 3.20 ± 1.47 µatm year^-1^ for the northwestern GoM and 2.35 ± 0.82 µatm year^−1^ for the west Florida Shelf. However, surface CO_2_ in the central (coastal and open) GoM, which is influenced by Mississippi and Atchafalaya River outflow, remained fairly stable over this time period.

## Introduction

Since the onset of the Industrial Revolution, atmospheric carbon dioxide (CO_2_) has increased by approximately 40% ^1^. The ocean is responsible for absorbing ~ 25% of anthropogenic CO_2_ emissions, which modulates climate by reducing global warming^1^. However, oceanic absorption of atmospheric CO_2_ also leads to an increase in seawater partial pressure of CO_2_ (pCO_2_), and a decrease in seawater pH and calcium carbonate (CaCO_3_) saturation state (Ω)^2,3^. This process, termed ocean acidification, has numerous negative consequences for marine organisms, especially those that form CaCO_3_ skeletons and shells (e.g. corals and shellfish), including a reduction in biogenic calcification and an increase in CaCO_3_ dissolution^4–8^.

Gulf of Mexico (GoM) ecosystems support fisheries that are essential to the Gulf Coast and U.S. economies^9^. The 2015 GoM seafood landings revenue was $858 million, which is almost 20% of the total U.S. landings revenue^9^. Despite serving as a critical component for regional economic stability, the GoM is subject to numerous anthropogenic pressures that contribute to the decline of ecosystem health, including eutrophication, hypoxia, oil spills, warming, and acidification^10–12^. Hypoxia in the northern GoM is driven by the spring flux of nutrients from the Mississippi and Atchafalaya River systems, combined with water column stratification that prevents mixing of bottom waters with oxygenated surface water^13–15^. The production of CO_2_ through respiratory processes also lowers bottom water pH (i.e., increases acidity)^11^. Hypoxia and associated bottom water acidification are predicted to intensify due to increased nutrient loadings and organic matter production^16,17^. Decadal declines in subsurface (100–250 m) Ω and pH have been reported on the outer continental shelf region, and are attributed to uptake of anthropogenic CO_2_ and elevated respiration^18^. Therefore, hypoxia and ocean acidification are highly relevant stressors for GoM ecosystem management.

In an effort to characterize GoM carbonate chemistry trends, several studies involving both observational and model data have been conducted^19–27^. Much of the carbonate chemistry work done thus far has focused on northern GoM continental shelf waters, where Mississippi and Atchafalaya River outflow and seasonal bottom water hypoxia occur^21,23,24^. Surface CO_2_ is elevated immediately adjacent to these river mouths due to the high pCO_2_ (~ 2000 µatm) of river water combined with turbidity that blocks sunlight and inhibits photosynthesis^23^. On the inner shelf, mixing of freshwater and seawater drives outgassing of CO_2_^23,28,29^. Riverine nutrients enhance productivity further lowering CO_2_ and dissolved inorganic carbon (DIC). Shelf surface water CO_2_ and DIC are therefore lower than offshore levels, with the lowest concentrations observed on the Louisiana shelf near the Mississippi and Atchafalaya River outflow^22^. Total alkalinity (TA) (mostly in the form of bicarbonate) in the freshwater end-member is highly variable, and depends on the volume of river discharge available to dilute the weathering signal produced by the dissolution of continental rock minerals, which varies seasonally^28–30^. Photosynthetic drawdown of shelf surface water DIC increases TA:DIC (to approximately 1.24), aragonite saturation state (Ω_ar_; to > 5) and the buffering capacity of Louisiana shelf water^22,25^. In addition, seasonal CO_2_ trends have been identified on the shelf. The northern GoM and west Florida shelves are sinks for atmospheric CO_2_ in the winter and spring, and sources of atmospheric CO_2_ in the summer and fall due to seasonal cycles in nutrient loading and temperature^26,31^. The western GoM shelf is less influenced by fluvial nutrient inputs from the Mississippi and Atchafalaya rivers and serves as a CO_2_ source for most of the year, except during winter when temperatures are low^27^.

Compared to coastal waters, GoM open ocean carbonate chemistry is fairly stable. Temperature drives CO_2_ variability and the open ocean has been classified as a sink for atmospheric CO_2_ for most of the year, except during the summer when temperatures are high^27,32^. The Loop Current originates in the Caribbean, enters the GoM through the Yucatan Strait, extends north toward the shelf and exits through the Straits of Florida. This warm water current delivers relatively low pCO_2_ and DIC, and high TA water into the GoM^19,22^.

Although regional and seasonal GoM carbonate chemistry trends have been identified, there are also some discrepancies across studies. For example, Chavez et al.^33^ classified the entire GoM as a net annual CO_2_ source to the atmosphere, while more recent studies characterize the GoM as an annual CO_2_ sink^27,32,34^. Xue et al.^27^ and Huang et al.^23^ found the northern GoM shelf serves as an annual CO_2_ sink but Xue et al.^27^ showed strong, cross-shelf variations that were linked to seasonal and spatial salinity gradients. In addition, the coupled physical-biogeochemical model developed by Xue et al.^27^ simulated higher summer pCO_2_ values on the northern GoM shelf than are shown in the observational data. Differences across studies are likely the result of the dynamic physical (e.g. shelf circulation) and biological (e.g. organic metabolism) controls on coastal pCO_2_, which introduce variability on multiple time and space scales.

The objective of this paper is to synthesize publicly available, underway surface seawater CO_2_ data in the GoM from 1996 to 2017, in order to investigate spatial, seasonal, and long-term basin-wide trends. This analysis avoids the spatial and temporal limitations of subregion-specific observational studies while still allowing for detailed assessments over small spatial scales. The limitations of this dataset include a bias towards spring and summer observations; incomplete spatial coverage in the western GoM and on the West Florida and Mexican continental shelves; and limited spatial and temporal overlap between datasets (i.e., there are temporal gaps in the dataset and unlike long-term time-series programs, repeat sampling did not occur in the same location(s) over time). Nevertheless, this study provides the most comprehensive description of GoM spatiotemporal CO_2_ trends to date and allows for the examination of surface CO_2_ system dynamics at a regional scale over more than two decades. As more CO_2_ measurements are collected, it will be important to update this analysis in order to assess the GoM CO_2_ system response to regional and global perturbations.

## Results

**Figure 1:**
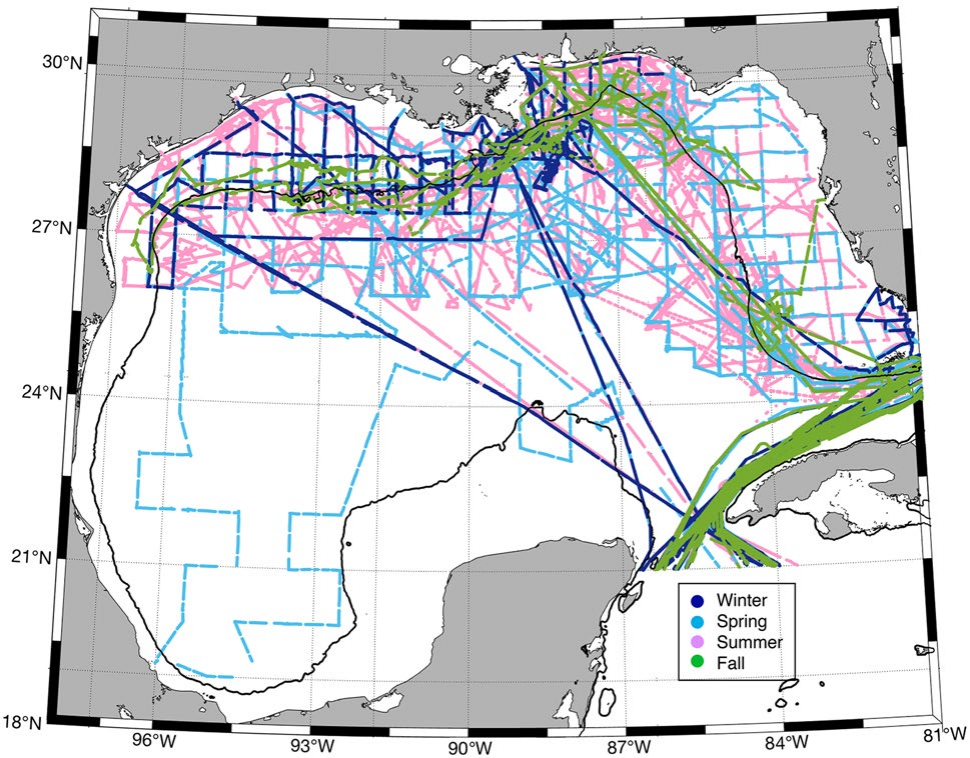
Approximately 381,000 underway measurements were collected between 1996 and 2017. Measurements are colored according to season: winter (dark blue), spring (light blue), summer (pink) and fall (green). The solid black line represents the 200 m bathymetry contour.

Approximately 380,000 underway measurements of sea surface temperature (SST), sea surface salinity (SSS) and surface seawater CO_2_ within the GoM spanning 1996–2017 were obtained from the Surface Ocean CO_2_ Atlas (SOCAT). The data were separated into regions of the coastal (0–200 m depth) and open ocean GoM (> 200 m depth, i.e. beyond the continental shelf). For long-term trend analyses and air-sea CO_2_ flux calculations, the data were further subdivided into three coastal regions and six open ocean regions (see Methods and Figure S1). There are approximately 138,500 coastal measurements and 242,300 open ocean measurements and the majority of data (> 99%) were collected after 2001. Summer (Jun–Sep: ~ 166,000) and spring (Mar–May: ~ 117,000) seasons account for 75% of the dataset with far fewer measurements collected during the winter (Dec–Feb: ~ 56,000) and fall (Oct–Nov: ~ 42,000) seasons (Fig. 1 and Table S1), leading to potential biases in the annual flux calculations and seasonal cycle characterizations. Additional future measurements will aid in verifying and updating the annual fluxes, and winter and fall trends reported in this study.

### Open Ocean GoM

**Figure 2:**
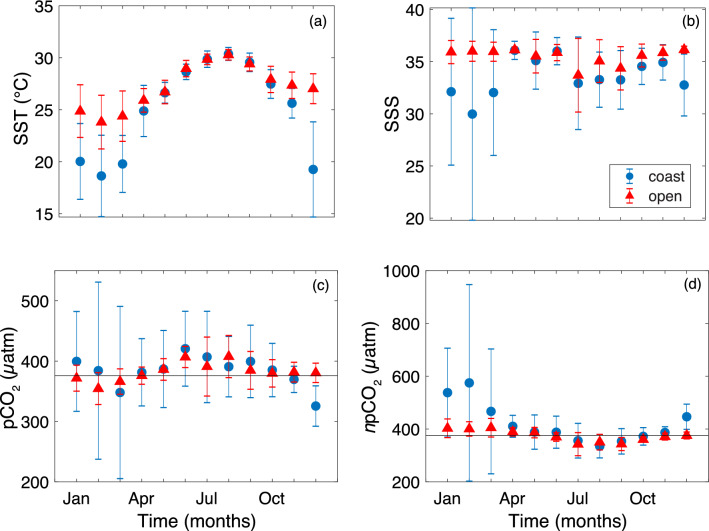
Monthly mean sea surface temperature (SST in °C) (**a**), sea surface salinity (SSS) (**b**), sea surface partial pressure of CO_2_ (pCO_2_ in μatm) (**c**) and temperature-normalized sea surface pCO_2_ (*n*pCO_2_ in μatm) (**d**). Coastal means (± std) are represented by the blue circles and open ocean means (± std) are shown by the red triangles. The solid lines represent mean atmospheric CO_2_ concentration (376 ± 6 μatm) over the study period.

**Figure 3:**
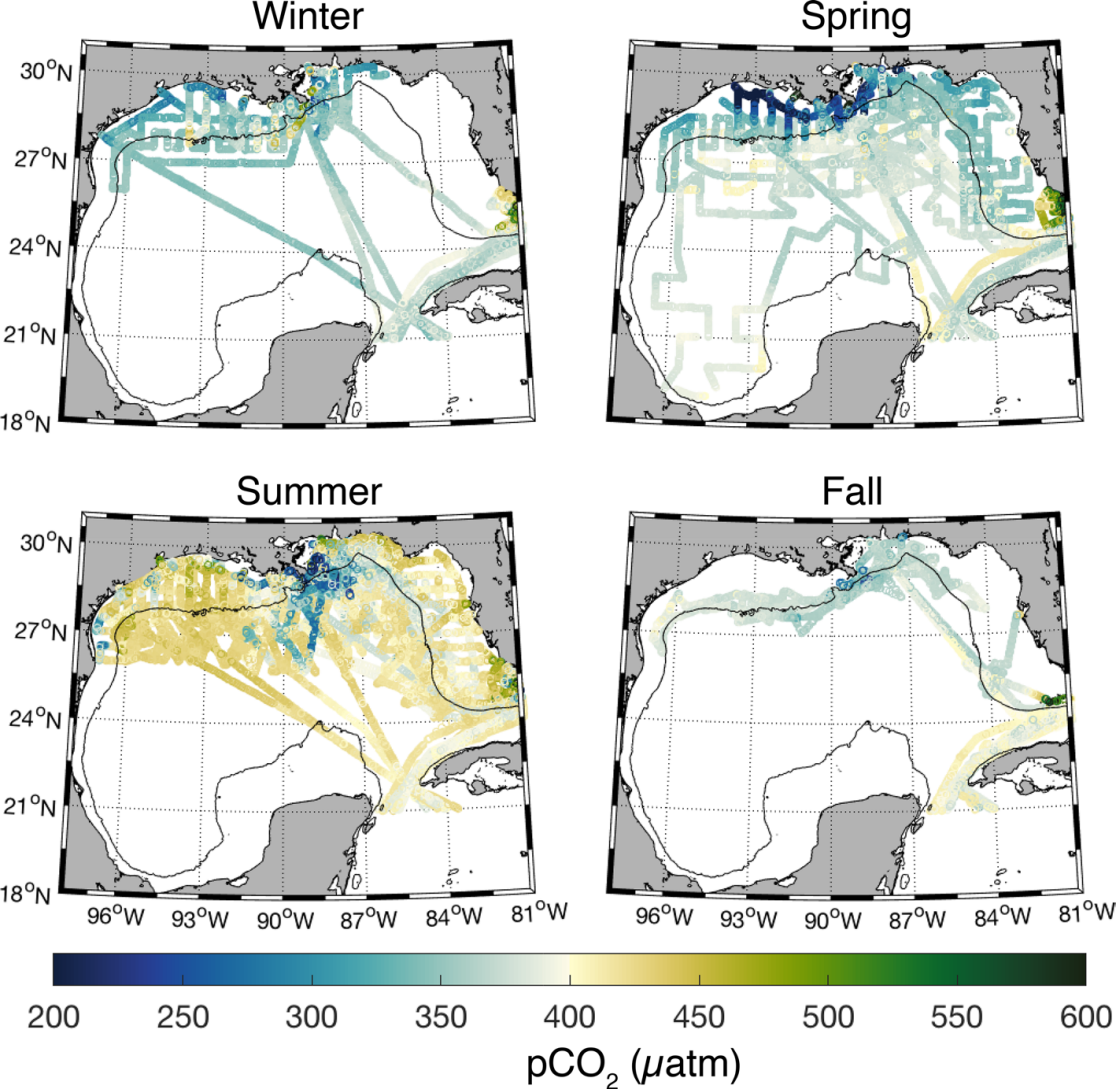
Surface seawater CO_2_ partial pressure (pCO_2_ in μatm) during winter, spring, summer and fall. Note that the actual minimum and maximum values are 69 and 1784 µatm, respectively. The solid black line represents the 200 m bathymetry contour.

Open ocean SSTs ranged from 13.4 °C to 32.2 °C, with the lowest mean SSTs occurring in winter (Dec–Feb) to early spring (Mar–May) (Feb mean SST = 23.8 ± 2.6 °C; means presented as the average of all data collected during each month or season ± one standard deviation throughout) and maximums occurring in the summer (Jun-Sep) (Aug mean SST = 30.3 ± 0.5 °C) (Figs. 2 and S2; colormaps herein from Thyng et al.^35^). Average open ocean SSS was 35.3 ± 1.9 (range = 18.9–37.2) (Figs. 2 and S3). Monthly averaged salinity was fairly constant, except in July when the mean value decreased to 33.7 ± 3.5. The seasonal cycle of open ocean pCO_2_ (range = 124–493 µatm) mirrored SST, with lower levels in the winter (Feb mean pCO_2_ = 354 ± 26 µatm) and higher levels in the summer (Aug mean pCO_2_ = 408 ± 35 µatm) (Figs. 2 and 3). Open ocean pCO_2_ normalized to an annual mean temperature (*n*pCO_2_) ranged from 122–630 µatm, with the lowest monthly means in summer (Jul mean *n*pCO_2_ = 343 ± 44 µatm) and the highest monthly means in late winter through early spring (Mar mean *n*pCO_2_ = 405 ± 35 µatm) (Fig. 2 and S4).

**Figure 4:**
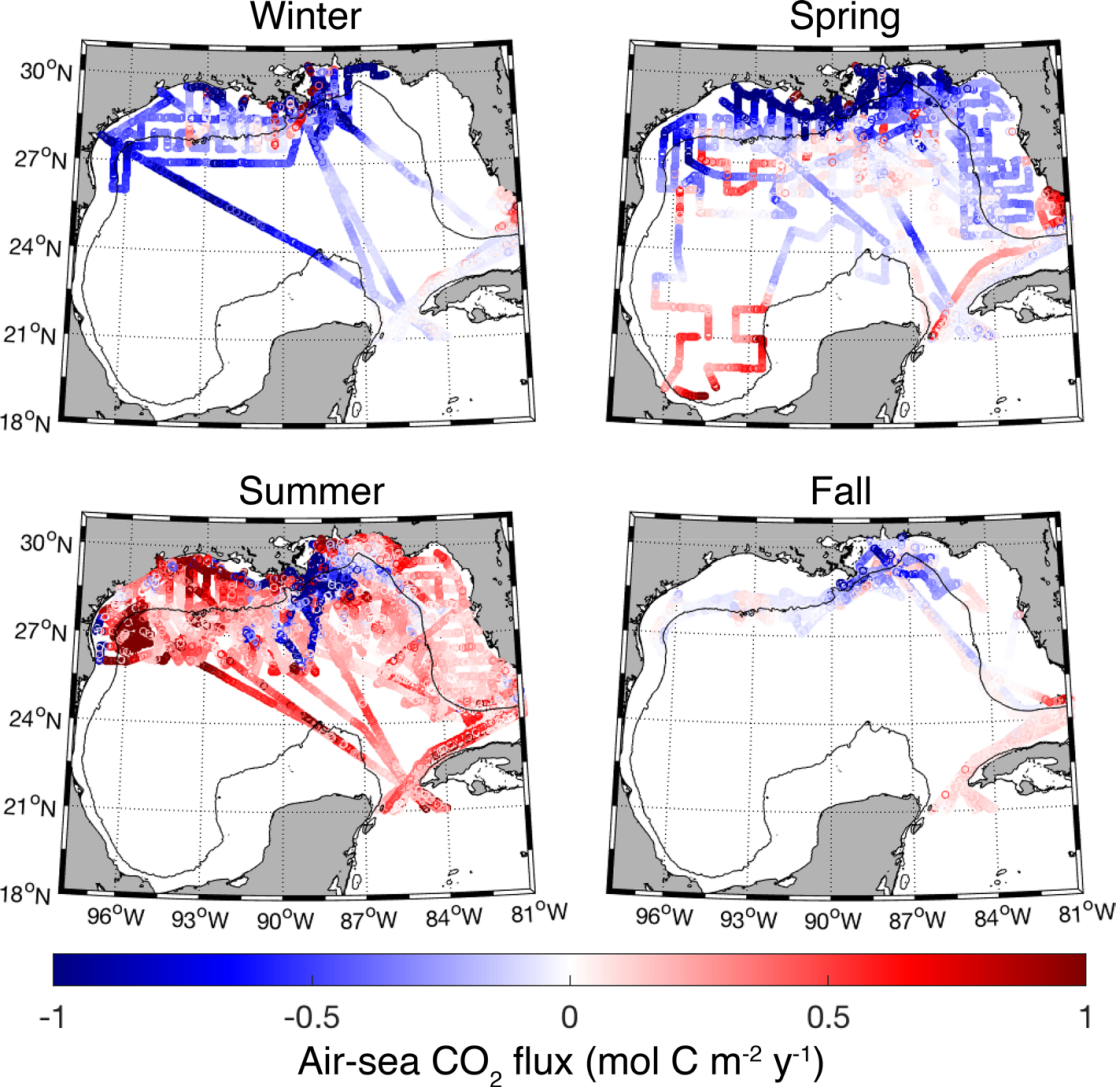
Seasonal air-sea CO_2_ fluxes (mol C m^−2^ year^−1^) calculated using the wind speed parameterization of Ho et al.^36^. Note that the actual minimum and maximum values are approximately − 30 and 39 mol C m^−2^ year^−1^, respectively. Positive values (red symbols) indicate an oceanic CO_2_ source to the atmosphere and negative values (blue symbols) indicate oceanic uptake of CO_2_. The solid black line represents the 200 m bathymetry contour.

Open ocean air-sea CO_2_ fluxes for both wind speed parameterizations^36,37^ agreed well (fluxes reported in the text and Table 1 are calculated using the parameterization of Ho et al.^36^ and fluxes for both parameterizations are shown in Figure S5). Since the majority of measurements were collected in the spring and summer, calculating the average annual flux based on all data introduces a seasonal bias. Hence, the average annual flux was calculated from the mean seasonal fluxes shown in Table 1. Open ocean air-sea CO_2_ fluxes ranged from − 7.71 to 6.03 mol C m^−2^ year^−1^ with an annual mean of − 0.06 ± 0.45 mol C m^−2^ year^−1^, indicating that the open ocean shows some seasonal variability, but is roughly balanced in terms of source and sink characteristics on an annual basis (Figs. 4 and S6; Table 1). The open ocean was a summer source (0.15 ± 0.58 mol C m^−2^ year^−1^) and a winter sink (− 0.32 ± 0.73 mol C m^−2^ year^−1^) for atmospheric CO_2_, whereas spring and fall fluxes were close to balanced and showed less variability than in summer and winter (Table 1). Spatially, most of the open ocean GoM (excluding the southwest and southcentral GoM, which did not contain enough data to determine annual fluxes) was roughly balanced in terms of being an annual source or sink, with the exception of the central GoM, which was a weak annual sink for CO_2_ (− 0.28 ± 0.58 mol C m^−2^ year^−1^) (Figure S6).

**Table 1:** Seasonal and annual means ± standard deviations of air-sea CO_2_ fluxes (mol C m^−2^ year^−1^) for the open and coastal ocean using the wind speed parameterizations of Ho et al.^36^.

Season	Mean	Min	Max
**Open ocean**			
Winter	− 0.32 ± 0.73	− 0.58 ± 1.03 (Feb)	0.04 ± 0.15 (Dec)
Spring	− 0.03 ± 0.29	− 0.19 ± 0.27 (Mar)	0.07 ± 0.31 (May)
Summer	0.15 ± 0.58	0.01 ± 0.38 (Sept)	0.35 ± 0.70 (Aug)
Fall	− 0.04 ± 0.18	− 0.05 ± 0.19 (Oct)	− 0.03 ± 0.16 (Nov)
Annual	− 0.06 ± 0.45		
**Coastal ocean**			
Winter	0.23 ± 4.68	− 0.47 ± 0.26 (Dec)	0.62 ± 7.10 (Feb)
Spring	− 0.31 ± 1.62	− 0.62 ± 2.45 (Mar)	− 0.08 ± 0.65 (May)
Summer	0.11 ± 0.63	0.09 ± 0.55 (Aug)	0.21 ± 0.25 (Jun)
Fall	− 0.15 ± 0.37	− 0.17 ± 0.38 (Nov)	− 0.09 ± 0.33(Oct)
Annual	− 0.03 ± 1.83		

For each season, the minimum and maximum monthly averages are also presented. Positive fluxes indicate the ocean is a CO_2_ source to the atmosphere while negative values indicate the ocean is a CO_2_ sink.

**Figure 5:**
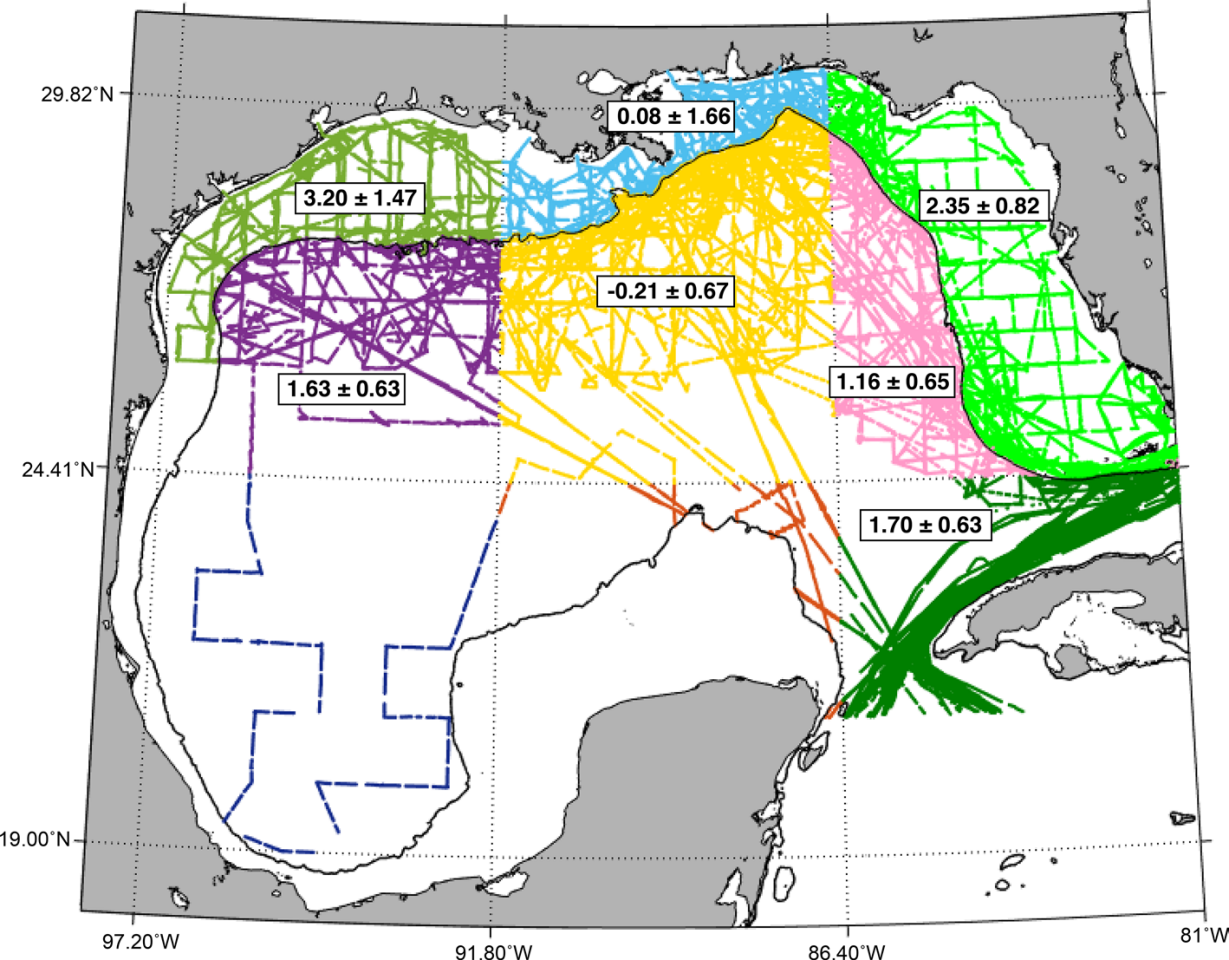
Long-term deseasonalized CO_2_ partial pressure (pCO_2_) trends (µatm year^−1^) across 7 sub-regions of the Gulf of Mexico. The black solid line represents the 200-m bathymetry line and separates the coastal and open oceans.

The long-term trends in deseasonalized open ocean pCO_2_ are 1.63 ± 0.63 µatm year^−1^ for the northwest, − 0.21 ± 0.67 µatm year^−1^ for the central, 1.16 ± 0.65 µatm year^−1^ for the northeast, and 1.70 ± 0.14µatm year^−1^ for the southeast GoM (Fig. 5 and S7, Table S2). Excluding the central open ocean, the overall average pCO_2_ increase in the remaining open ocean regions (i.e. northwest, northeast, southeast) is 1.50 ± 0.47 µatm year^−1^ (Fig. 5 and S7, Table S2). The southwest and southcentral open ocean GoM do not have enough data to observe seasonal or long-term trends.

### Coastal ocean

With the exception of December, the monthly means of GoM open and coastal SST, SSS, and pCO_2_ were not statistically different (Fig. 2). However, coastal SST, SSS and pCO_2_ were approximately two times more variable than the open ocean GoM. Coastal SSTs ranged ~ 8–33 °C, with the lowest mean value in February (18.6 ± 3.9 °C) and the highest mean value in August (30.4 ± 0.6 °C) (Figs. 2 and S2). Coastal ocean salinities ranged 0–40.3 along the shelf and minimum values were recorded near river mouths, particularly the Mississippi and Atchafalaya (Figs. 2 and S3). Monthly mean salinity was greatest in Apr–Jun and lowest in Dec–Mar (Figs. 2 and S3). The range for pCO_2_ in the coastal region was 69–1784 µatm, with the lowest mean monthly values in the winter (Dec mean = 326 ± 33 µatm) and highest mean monthly values in the summer (Jun mean = 420 ± 62 µatm) (Figs. 2 and 3). When coastal ocean pCO_2_ is normalized to an annual mean temperature (i.e., *n*pCO_2_; range = 66–3,090 µatm), the upper limit increases due to the removal of the dampening effect of low winter temperatures on pCO_2_. Maximum coastal pCO_2_ and *n*pCO_2_ values were recorded on the west Florida Shelf (WFS) and in the river mouths. Lowest coastal ocean average *n*pCO_2_ values occurred in the summer in the northern Gulf, particularly in August (335 ± 44 µatm), and maximum average *n*pCO_2_ values were in the winter (Feb mean = 575 ± 372 µatm) (Figs. 2 and S4).

The annual mean air-sea CO_2_ flux of the coastal ocean was slightly negative (mean = -0.03 ± 1.83 mol C m^−2^ year^−1^) (Table 1) with a large range and variability (− 29.91 to 38.29 mol C m^−2^ year^−1^) when compared to the open ocean (Fig. 4 and Table 1). The coastal ocean was a sink for CO_2_ during spring and fall (− 0.31 ± 1.62 and − 0.15 ± 0.37 mol C m^−2^ year^−1^, respectively) and a source in the winter and summer (0.23 ± 4.68 and 0.11 ± 0.63 mol C m^−2^ year^−1^, respectively). Waters immediately adjacent to major river mouths (Mississippi and Atchafalaya) were a strong, year-round source of CO_2_ to the atmosphere (mean = 7.30 ± 11.75 mol C m^−2^ year^−1^, defined as waters with salinity less than 17) but cover a small geographic area of the GoM coastal ocean. Spatially, the northwestern GoM (NW GoM; − 0.22 ± 0.59 mol C m^−2^ year^−1^) and northcentral GoM (NC GoM: − 0.25 ± 2.93 mol C m^−2^ year^−1^) were small annual CO_2_ sinks, while the WFS (0.07 ± 0.35 mol C m^−2^ year^−1^) was approximately balanced (Figure S6). The long-term trend in deseasonalized coastal pCO_2_ was 3.20 ± 1.47 µatm year^−1^ for the NW GoM, 0.08 ± 1.66 µatm year^−1^ for the NC GoM and 2.35 ± 0.82 µatm year^−1^ for the WFS (Fig. 5 and S8, Table S2).

### EOF analysis

**Figure 6:**
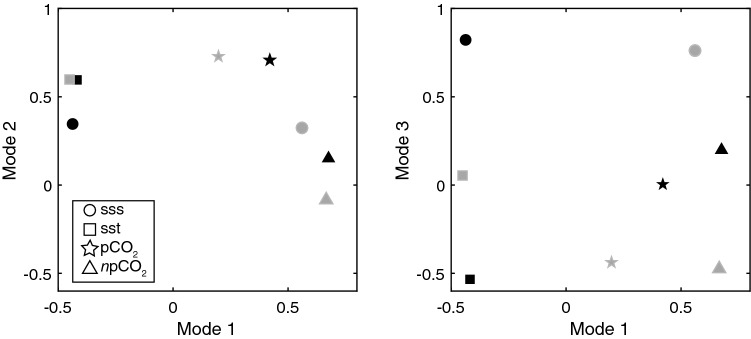
EOF analysis of Mode 1 and Mode 2 (left panel) and Mode 1 and Mode 3 (right panel) for the coastal ocean (black symbols) and open ocean (gray symbols). The four parameters of the EOF analysis include sea surface salinity (sss, circles), sea surface temperature (sst, squares), sea surface partial pressure of CO_2_ (pCO_2_, stars) and temperature-normalized sea surface pCO_2_ (*n*pCO_2_, triangles).

The results of the EOF show that about half of the observed variance in the open ocean dataset is attributed to Mode 1, Mode 2 represents 40% of the variance, and Mode 3 accounts for 8%. Because of this distribution, only Modes 1 and 2 of the open ocean data decomposition are likely statistically significant^38^. Furthermore, the dataset is temporally biased to include more spring and summer measurements, and spatially biased toward the northern and southeastern GoM. Hence, the EOF results are heavily influenced by these seasons and the local processes that dominate these regions. Based on examination of the magnitudes of the eigenvectors (Fig. 6, gray symbols), SSS and *n*pCO_2_ are positively correlated to each other and are strongly tied to Mode 1; SST is anti-correlated (i.e., of opposite sign) to SSS and *n*pCO_2_, but is of about equal magnitude. pCO_2_ variability is weakly accounted for in Mode 1. It is therefore possible that the open ocean Mode 1 represents a biological component linked to river discharge when warm, low salinity and low *n*pCO_2_ waters derived from the coast in the summer are advected into the oligotrophic open ocean, which is salty and comparatively cool. A map showing amplitudes of Mode 1 for each observation location highlights the relationship of Mode 1 to the proximity of the Mississippi River delta in the northern GoM near 90°W (see darker greens and blues near MS river delta in Figure S9, left panel).

SST and pCO_2_ load high, 0.55 and 0.75, respectively, for open ocean Mode 2 (Fig. 6, gray symbols), while SSS loading is relatively small (0.4) and *n*pCO_2_ is near zero. Examination of seasonal variability of Mode 2 shows a pronounced annual cycle that peaks in summer (June–August) and is of opposite sign in winter (Jan–Mar) (Figure S9: right panel). Therefore, the open ocean Mode 2 is likely tied to seasonal changes in temperature. Mode 3 is not a significant component of the open ocean EOF but the amplitudes show that this mode is dominated by SSS. To summarize, the EOF analysis shows that proximity to the Mississippi River plume accounts for more than half of the observed variance in the open ocean dataset and the remaining variance is accounted for by seasonal variations related to temperature.

In the coastal ocean, the EOF indicates that Mode 1 represents 51%, Mode 2 represents 31% and Mode 3 represents 17% of the observed variance in the data. Most of the coastal data were also collected in the northern GoM in spring and summer. Therefore, the coastal EOF conclusions are strongly influenced by the processes that dominate variability within these seasons and this region. Based on examination of the magnitudes of the eigenvectors (Fig. 6, black symbols), pCO_2_ and *n*pCO_2_ are positively correlated to each other and strongly tied to Mode 1. There is also a negative correlation of pCO_2_ and *n*pCO_2_ to SST and SSS (i.e., high CO_2_ implies low SST and low SSS and vice versa). There is a spatial pattern in the coastal ocean Mode 1 that highlights the regions near the Mississippi-Atchafalaya Rivers Galveston Bay, and Florida Bay to the south (red symbols, Figure S10, left panel). It is therefore likely that the coastal Mode 1 is linked to biological activity associated with river inputs. Initially, river water has high CO_2_, and low salinity and temperature. When mixed with shelf-water (higher salinity and temperature), degassing and photosynthesis decrease CO_2_ in these waters.

Coastal ocean Mode 2 variability is driven by SST and pCO_2_ (Fig. 6, black symbols). Mode 2 likely represents the seasonality of temperature and its thermodynamic effect on CO_2_. The coastal Mode 3 amplitude is dominated by the strong SSS value, ~ 0.8, and the spatial map of the Mode 3 amplitudes highlights (in dark green) the inner shelf in close proximity to Mississippi-Atchafalaya Rivers (Figure S10, right panel). Mode 3 therefore represents the importance of these rivers in driving the salinity variability on the Texas-Louisiana Shelf. The results of the coastal EOF indicate about half of the observed variance in this dataset can be attributed to riverine processes and associated biological activity, about a quarter of the variance can be attributed to seasonal temperature variations, and about a quarter of the observed variance can be attributed to salinity variability associated specifically with the Mississippi and Atchafalaya Rivers.

We also examined the EOF results to investigate how the forcings of variability change over time. The open ocean data were separated into two temporal groups: 2007–2012 and 2013–2017. Data before 2007 were excluded due to limited temporal and spatial coverage in the earlier years. Regardless of time, the main driver of open ocean variability (i.e., Mode 1) is a biological component linked to river discharge and accounts for half of the variance in the dataset (Figure S11, Table S2). The coastal ocean data were grouped into temporal groups of 2003–2010 and 2011–2017. Biological production associated with river water remains the main driver (i.e., Mode 1) of variability over time, but its relative importance to the other modes increased slightly from 46% in the earlier years to 58% in the latter years (Figure S12, Table S4).

## Discussion

### Open ocean

Spatial and temporal trends in GoM open ocean CO_2_ are controlled by temperature, biological production, and physical forcings. Seasonal changes in temperature dominate the pCO_2_ seasonal cycle with lower pCO_2_ in the winter and higher pCO_2_ in the summer (Fig. 2). This trend is a result of the effect of temperature on CO_2_ solubility (i.e., higher temperature decreases CO_2_ solubility). However, the summer warming effect on open ocean CO_2_ is partially offset by photosynthetic drawdown of CO_2_ as shown by the winter-to-summer decrease of approximately 50 µatm in *n*pCO_2_ (Fig. 2).

**Figure 7:**
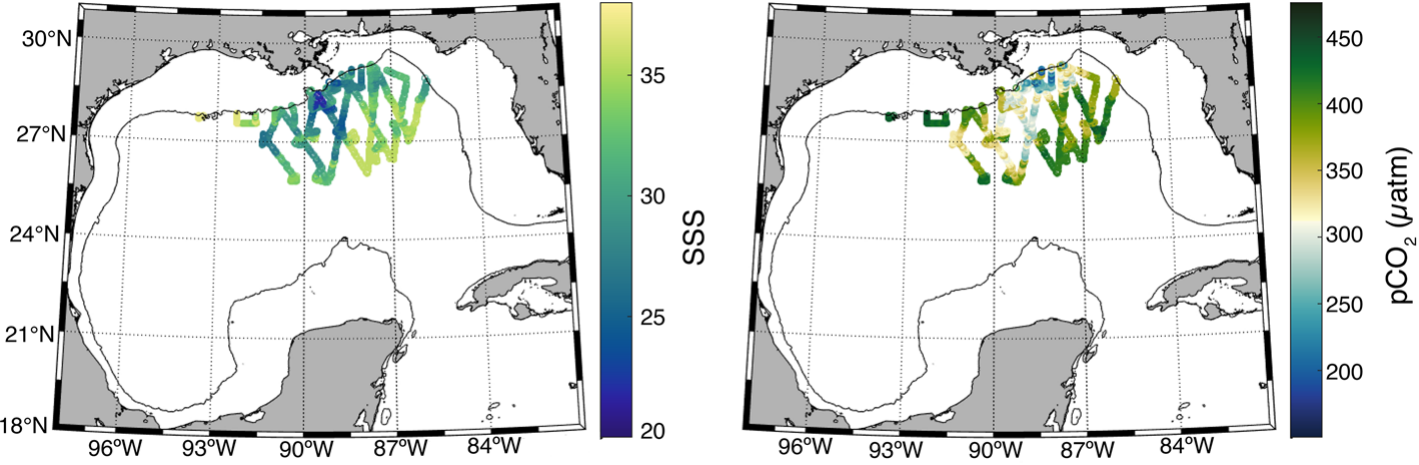
Open ocean sea surface salinity (left panel) and surface seawater partial pressure of CO_2_ (μatm) (right panel) during July 2009. The solid black line represents the 200 m bathymetry contour.

The EOF analysis suggests that organic production associated with freshwater input is the primary driver of variability in the open ocean dataset (Figure S9). This is due to cross-shelf transport events like the one that occurred in July 2009^24,39–42^, when low pCO_2_ and low salinity water extended from the coast, beyond the shelf break, and greater than 300 km offshore (Fig. 7). River flow during 2009 was higher than average (220,000 m^3^ s^−1^ compared to an annual average of 174,000 ± 32,800 m^3^ s^−1^ (https://rivergages.mvr.usace.army.mil/; station 01100Q)). Persistent winds drove coastal surface currents and the river plume upcoast (eastward) and offshore (https://pong.tamu.edu; https://tabs.gerg.tamu.edu, Buoy R) (Figure S13), causing low-salinity and low CO_2_ freshwater discharge to pool within the north central and northeastern GoM^43,44^. Sea surface height anomalies obtained from the Colorado Center for Astrodynamics Research (CCAR) reveal two cyclonic eddies located in the northeastern GoM during July 2009 (Figure S14). These eddies, which have − 30 cm and − 20 cm sea surface height anomalies at the eddy core, are located along the boundary of the prominent anticyclonic eddy shedding off the Loop Current. As these eddies impinge on the continental shelf, they enhance cross-shelf exchange by transporting coastal waters seaward^45,46^. Cross-shelf transport of nutrient-rich coastal waters in July 2009 also led to anomalously high chlorophyll and organic matter concentrations, and low DIC in the oligotrophic open ocean^24,40,41^.

Open ocean GoM waters are typically a summertime source of CO_2_ to the atmosphere, but this combination of physical processes can cause tens of thousands of square kilometers of the open ocean GoM to serve as a CO_2_ sink. Underway data in this study show average open ocean pCO_2_ during July 2009 (362 ± 56 µatm) is lower than for all months of July combined (391 ± 48 µtam), though not significantly different (Fig. 2). The patches of low pCO_2_ and low salinity water in the area persisted through August and dissipated by October, indicating that the effects of these combined processes (high river flow, offshore currents, and eddy activity) on open ocean surface water CO_2_ took about 1–2 months after peak spring river flow to develop and lasted approximately three months. Processes that enhance exchange at the shelf break can therefore transport low salinity, low CO_2_ and potentially nutrient-rich water associated with the Mississippi-Atchafalaya Rivers hundreds of kilometers offshore, enhance CO_2_ variability in the open ocean, and have the potential to impact north-central GoM open ocean ecosystems.

The chemical characteristics and spatial extent of the Loop Current also influence open ocean CO_2_ variability. Xue et al.^27^ classified the Loop Current as a strong, year-round CO_2_ sink. In contrast, underway CO_2_ measurements in this study show that waters within and just north of the Yucatan Strait are a weak annual source of CO_2_ to the atmosphere (Figure S6). Intrusion of the Loop Current into the northern GoM can also entrain Mississippi-Atchafalaya River discharge, enhance cross-shelf exchange and transport low CO_2_ and low DIC coastal water to the interior basin and out through the Florida Strait. Hence, spatial variability in the extension of the Loop Current as well as seasonal and interannual carbonate chemistry variability on the shelf, will influence the GoM open ocean inorganic carbon budget and the source water supplied to coral reefs of the Florida Reef Tract.

Air-sea flux estimates from this study indicate the open ocean GoM is approximately neutral in terms of an annual source or sink for atmospheric CO_2_ (− 0.06 ± 0.45 mol C m^−2^ year^−1^, Table 1). Model simulations showed the GoM open ocean is a strong annual CO_2_ sink (− 1.04 ± 0.46 mol m^−2^ year^−1^ C), particularly in the winter (− 2.44 ± 0.49 mol m^−2^ year^−1^ C)^27^ while Robbins et al.^34^ classified the open ocean GoM as a much smaller annual CO_2_ sink (− 0.48 ± 0.07 mol m^−2^ year^−1^ C). Discrepancies in the magnitude of the fluxes may result from interannual variability in cross-shelf events, differences in wind speed averages (10-day intervals in^27^, monthly mean winds in Robbins et al.^34^, and daily average wind speeds here), binning and averaging of CO_2_ data, time span of the dataset analyzed, and spatial or seasonal sampling biases.

### Coastal ocean

The main drivers of spatiotemporal variability of coastal surface seawater CO_2_ are biological production, which can be associated with river discharge, temperature, and coastal water circulation. Nutrient loading enhances coastal biological production in the spring and results in low CO_2_ on the northern GoM shelf despite increasing SSTs^22,28,29^ (Figs. 2 and 3). High summer SSTs cause coastal CO_2_ to increase in most areas, except in the north central GoM, which is likely due to the residual effects of spring discharge and subsequent biological production. Low temperatures resulting from cooling of shallow coastal waters, the migration of cold fronts from the north that extend over the coastal region, and/or mixing with cold, freshwater outflow, cause coastal CO_2_ to decline in the winter.

Surface currents play a key role in determining the spatial extent of Mississippi and Atchafalaya discharge and its influence on GoM coastal carbonate chemistry. The effect of coastal circulation on CO_2_ variability on seasonal time scales is apparent through the salinity distribution over the coastal shelf during the spring and summer (Figure S3). Although river discharge peaks in the spring, the influence of these low salinity waters persists for several months. Downcoast (toward the west) currents from fall through spring carry river discharge along the inner shelf of the northwestern Gulf^47^. In the spring, surface water CO_2_ is low in these nutrient-rich, freshwater plumes due to elevated organic productivity (Figs. 3 and S4). When the winds shift eastward in the summer, coastal currents drive oligotrophic open ocean waters onto the northwestern shelf^44^. Westward flow of river discharge is restricted by the coastline causing these productive, low CO_2_ waters to pool on the central and northeastern GoM shelves and even be transported offshore.

In general, coastal waters are a sink for atmospheric CO_2_ during the fall and spring due to cooler temperatures and enhanced biological productivity, respectively, and a CO_2_ source during the summer and winter due to higher temperatures and enhanced respiration, respectively (Figs. 4 and S4, Table 1). The NW GoM and NC GoM were annual sinks, while the WFS was a weak annual source. These results are in general agreement with Xue et al.^27^ although some differences do exist. For example, Xue et al.^27^ characterizes the western coastal shelf as an annual CO_2_ source and the northern Gulf and WFS as a stronger annual sink and source, respectively. Robbins et al.^31^ classified the WFS as a small annual net source of CO_2_, which is in agreement with this study (Figure S6).

### Long term trends

The underway CO_2_ data presented here span a sufficient length of time to detect changes in open ocean carbonate chemistry due to oceanic uptake of anthropogenically produced atmospheric CO_2_^48–51^. However, unlike long-term time-series programs such as the Hawaii Ocean Time-series (HOT) in the Pacific, the Bermuda Atlantic Time-series Study (BATS) in the Atlantic, and others, this dataset is inconsistent across time and space. In addition, the long-term trend analysis is calculated from an ~ 14 year time-series (rather than the full 1996–2017 period) for three of the four open ocean bins since data are not available for those bins until 2003 (Figures S7). It is therefore possible that the long-term trends in bins with shorter time-series are influenced by climatic oscillations that occur on decadal time scales, rather than by anthropogenically-driven change^52^. Despite the non-ideal nature of this dataset for examining interannual trends, there is evidence for anthropogenic ocean acidification in the GoM open ocean. In the northwest and northeast open ocean GoM, pCO_2_ is increasing at 1.63 ± 0.63 µatm year^−1^ and 1.16 ± 0.65 µatm year^−1^ (Table S2 and Fig. 5), respectively, with no significant long-term trends in SST, SSS or *n*pCO_2_ (though *n*pCO_2_ is increasing). These increasing pCO_2_ trends are comparable to the rates measured at HOT (1.72 ± 0.09 µatm year^−1^) and BATS (1.69 ± 0.11 µatm year^−1^) and are likely due to oceanic uptake of anthropogenic CO_2_ from the atmosphere^49^. pCO_2_ in the southeast open ocean GoM is increasing at a similar rate (1.70 ± 0.14 µatm year^−1^) as the northwest and northeast. However, approximately 25% of this trend can be attributed to rising SSTs (0.03 °C year^−1^, *p* < 0.01, R^2^ = 0.14)^53^ and the remaining pCO_2_ increase (i.e., *n*pCO_2_) (1.19 µatm year^−1^, *p* < 0.01, R^2^ = 0.50) is likely due to uptake of anthropogenic atmospheric CO_2,_ a shift towards net heterotrophy, or a combination of both. There are no long-term trends in pCO_2_, SST, SSS, or *n*pCO_2_ in the central open ocean GoM, potentially because this region is influenced by Mississippi and Atchafalaya river waters, which affect surface water productivity and introduce variability that may mask long-term trends. The establishment of a GoM open ocean time series station that includes carbonate chemistry measurements is needed to elucidate long-term ocean acidification trends with better certainty and to understand how this global phenomenon will impact the health of GoM coral reefs and fisheries.

Surface water pCO_2_ is increasing faster in the coastal NW GoM (3.20 ± 1.47 µatm year^−1^) and WFS (2.35 ± 0.82 µatm year^−1^), than in the open ocean (Fig. 5 and Table S2). Since there are no significant SST or SSS trends in the NW GoM, the enhanced acidification signal may be driven by a decline in the photosynthesis to respiration ratio and/or uptake of anthropogenic atmospheric CO_2_. On the WFS, SST is increasing 0.07 ± 0.04 °C year^−1^ (R^2^ = 0.09, *p* = 0.06) and accounts for ~ 40% of the WFS pCO_2_ rise^54^. The remaining 60% (or 1.35 µatm year^−1^) is similar to the open ocean pCO_2_ trend and may be due to absorption of atmospheric CO_2._ However, it is unclear why the WFS pCO_2_ rise is not also reflected in *n*pCO_2_ (Table S2). In contrast, there are no significant long-term trends in SST, SSS, pCO_2_ or *n*pCO_2_ in the NC GoM coastal ocean. The NC GoM coastal ocean is influenced by Mississippi and Atchafalaya river waters, making this region highly variable and productive. Although we did not find evidence for significant long-term changes in organic production (i.e., *n*pCO_2_) in the NC GoM, the coastal EOF suggests that biological productivity plays a stronger role in driving variaiblity in latter years of the dataset (Table S4). It is also possible that an increase in pCO_2_ is masked by increased photosynthesis driven by excess terrestrial nutrient inputs^55^, or that the time-series is not sufficiently long enough for the anthropogenic trend to emerge^56^. The NW GoM and WFS coastal pCO_2_ increases observed in this study are similar to long-term CO_2_ trends measured at the CArbon Retention In A Colored Ocean (CARIACO) time series station in the Cariaco Basin of the south Caribbean Sea^49^, in coral reef systems^58^, in a previous study on the WFS^31^, and on the southeastern U.S. coastal margin^57^ . Together, these studies suggest that the coastal oceans may be acidifying more rapidly than the open ocean. Hence, coastal ecosystems, which contain economically-important marine calcifiers such as coral reefs and shellfish, may be the first to suffer the negative consequences of ocean acidification.

## Methodology

### Database

To ensure the most comprehensive compilation of quality controlled data, continuous measurements of SST, SSS and surface seawater CO_2_ within the GoM were downloaded from SOCAT v6^59^ for the years 1996–2017. SOCAT reports CO_2_ data as fCO_2_, but also provides the molar fraction of CO_2_ (xCO_2_), sea surface salinity and temperature, and equilibrator pressure, which we used to calculate pCO_2_. All datasets with flags A, B, C and D were included in this analysis^59^. Prior to submission to SOCAT, the data are QC/QA’d by submitting groups and all flags A-D have a CO_2_ accuracy of better than ± 5 µatm^59^. These publicly available data were made possible by the hard work of many groups including^19,21–25,41,60^ and others. Cruise identifiers and principal investigators for our final dataset can be found in Table S5.

### Underway data

Although underway CO_2_ systems vary slightly across research vessels, there are general principles of operation and quality control procedures^61^. Seawater is drawn in from an intake port located 5 m below the sea surface and circulated through a chamber which allows for CO_2_ equilibration between the water and overlying air. To limit the effects of water vapor, the head space gas travels through a condenser and Nafion tube before the CO_2_ mole fraction is measured by an infrared gas analyzer (IRGA). In most cases, the IRGA is calibrated using four CO_2_ gas standards within the range of 200–450 ppm in order to verify an accuracy within ± 2 ppm. A typical sequence consists of 60 equilibrator samples, six atmospheric boundary layer samples and one set of calibration gases, each measured at 2-min intervals. pCO_2_ was calculated using SST measured at the intake port, temperature and pressure of the equilibrator, water vapor pressure, and atmospheric pressure^61^.

The calculation for air-sea CO_2_ flux (F) is given as: F = kα∆pCO_2_, where k is the gas transfer velocity, α is the solubility of CO_2_ in seawater at in situ temperature and salinity^62^, and ∆pCO_2_ is the seawater pCO_2_ minus the atmospheric pCO_2_. Positive (negative) F represents a CO_2_ flux from the ocean to the atmosphere (atmosphere to the ocean). Daily average winds speeds at each location were obtained from the Cross-Calibrated Multi-Platform (CCMP) Wind Vector Analysis Product v2, which are referenced to a height of 10 m. The wind speed parameterizations for k proposed by Ho et al.^36^ and Wanninkhof^37^ were used to calculate F to be consistent with previous studies that used Wanninkhof^37^ and to compare results between different parameterizations. Atmospheric CO_2_ data were obtained from the monthly CO_2_ record at the Mauna Loa Observatory (https://www.esrl.noaa.gov/gmd/ccgg/trends/data.html). Atmospheric CO_2_ data are initially reported as a mole fraction in dry air. These data were corrected to 100% humidity by computing the water vapor pressure at SST and SSS, then converting to pCO_2_ using the instantaneous pressure reported in the underway datasets^63^. Important to note is that there is only a single spring dataset in the southwestern GoM (18–25°N, 87–97°W) (Fig. 1), which means that the open ocean and coastal CO_2_ summer, fall, and winter flux estimates and CO_2_ trends reported in this study do not include this region and the spring flux estimates do not incorporate a comprehensive representation of this region. The southwestern GoM is one of the major gaps in our knowledge of the GoM CO_2_ budget. In addition, since Fig. 4 shows that the northcentral GoM has different magnitude and sometimes direction of flux than the surrounding GoM, we divided the coastal and open oceans into smaller subregions and calculated annual fluxes separately for each subregion (Figure S6).

To aid in examining pCO_2_ trends driven by processes other than changing temperature (e.g. biological productivity), pCO_2_ was normalized to a constant temperature (*n*pCO_2_). The effects of temperature on isochemical water conditions for a temperature range of 2–28 °C and salinity range of 34–36 is 0.0423 C^−1^ and is given by the equation: *n*pCO_2_ = pCO_2insitu_*exp(0.0423*(SST_mean_ − SST_insitu_)), where pCO_2insitu_ and SST_insitu_ are the measured values and SST_mean_ is the annual mean SST (26.08 °C) of the entire dataset^53,64^. This relationship has previously been used across the global surface ocean, including in the GoM^64,65^. Although summer SSTs in the GoM sometimes exceed 30 °C, in general, temperatures are within the range of 2–28 °C (Figure S2). However, coastal salinity is highly variable, particularly near the river mouths, and approximately 70% of the measurements fall outside the salinity range 34–36 (Figure S3). For a fixed TA and DIC, the pCO_2_ increase or decrease °C^-1^ at a salinity of 20 is about 70% of the change of a water sample with a salinity of 35. This introduces a ~ 1% error (or ~ 5 µatm) to the *n*pCO_2_ estimates, which is equal to the uncertainty of the underway pCO_2_ dataset used here. Furthermore, freshwater TA can be significantly different from open ocean TA. Therefore, although we have calculated *n*pCO_2_ for the entire dataset, care must be taken when interpreting absolute values in areas directly near river outflow (salinity < 20, or < 1% of the dataset). While important to consider, these confounding factors have no effect on the interpretation of overall trends in > 99% of the dataset.

In order to evaluate long-term trends, we followed the deseasonalization procedure described in Takahashi et al.^65^. First, outliers (greater than three standard deviations from the median) were removed for each parameter (i.e., pCO_2_, *n*pCO_2_, SST and SSS). Then, seasonal cycles were determined by calculating a monthly mean from the 20 year data composite and a 20 year annual mean was calculated from the monthly means (Figure S15). The difference between the monthly mean and the annual mean is the correction applied to the monthly mean of each individual year (i.e., the deseasonalization). The deseasonalization was performed for the coastal ocean (0–200 m) and open ocean (> 200 m), and assumes that the seasonal cycles and corrections do not change over the time period covered by the dataset. Further, since Figs. 3 and 4 suggest that the northcentral GoM has a different seasonal cycle than surrounding areas, we divided the coastal and open oceans into smaller subregions and performed the deseasonalization separately for each subregion (Figures S1, S7, S8 and S15). The coastal ocean was divided into three subregions: NW GoM, NC GoM, and WFS (Figures S1, S8 and S15). The open ocean was divided into six bins: southwest, southeast, northwest, central and northeast (Figures S1, S7 and S15). The southwest and southcentral open ocean bins do not have enough data to observe seasonal or long-term pCO_2_ trends. For the open ocean deseasonalization and long-term trend calculations, we first removed data from the anomalous year 2009 when low salinity and low pCO_2_ coastal waters were transported offshore into the northcentral and northeastern GoM. Following deseasonalization, the long-term trend analyses were calculated separately for each coastal and open ocean bin (except for the southwest and southcentral open ocean bins that lack data) (Table S2). Other than the central region, all other open ocean bins (southeast, northwest, northeast) have similar seasonal cycles and long-term pCO_2_ trends (Figs. 5 and S15, Table S2). Hence, we report results for each open ocean bin separately and also as an average of the southeast, northwest, and northeast bins.

### Empirical orthogonal function

A Principal Component Analysis (PCA), or equivalently Empirical Orthogonal Function (EOF) analysis^66^, decomposes the input data into a linear combination of statistically independent (orthogonal) basis functions. The basis functions are the eigenvectors of the covariance matrix of the input data and are referred to as the modes of variability. The amplitudes of the basis functions are estimated as the projections of the eigenvectors onto the original data set. The eigenvalues of the covariance matrix represent the fractional percent of variance each eigenvector represents in the original data set. The EOF amplitudes can be interpreted spatially and temporally to identify patterns that can be associated with process mechanisms. The number of independent modes, (i.e., eigenvectors), is limited to the number of variables used in the calculation, which in this case is four: SST, SSS, pCO_2_ and *n*pCO_2_. These four variables were chosen because they have the greatest potential to drive variability in this dataset, and may be linked to biogeochemical and physical processes. The analysis was performed separately on GoM coastal (0–200 m) and open ocean data (> 200 m) since drivers behind variability can be different in these regions. The results of the analyses show that despite the number of EOF modes being limited to four, discernable temporal and spatial patterns emerge that are interpretable as being related to seasonal variability and proximity to terrestrial freshwater sources. The interpretations are necessarily broad due to the limited number of variables. However, in future studies the addition of other variables (e.g., currents, nutrients and other carbonate parameters) will better focus the processes responsible for the variances.

## Supplementary information

Supplementary information

## Data Availability

The datasets used in this study are all publicly-available through SOCAT (https://www.socat.info), ESRL (https://www.esrl.noaa.gov) and CCMP (https://www.remss.com/measurements/ccmp/).
