# Supplementary material for: Surface Water CO2 variability in the Gulf of Mexico (1996–2017)
Source: Sci Rep. 2020 Jul 23;10:12279. doi: 10.1038/s41598-020-68924-0 (PMC7378240; doi:10.1038/s41598-020-68924-0)
Supplement: Supplementary file 1 — Supplementary information [file 41598_2020_68924_MOESM1_ESM.pdf]

Surface Water CO_2 Variability in the Gulf of Mexico (1996-2017)
Supplementary Figures

Andrea K. Kealoha*[1,2], Kathryn E.F. Shamberger[1], Steven DiMarco[1], Kristen Thyng[1], Robert Hetland[1], Derek P. Manzello[3], Niall Slowey[1], Ian Enochs[3]

1. Texas A&M University, Department of Oceanography, College Station, TX, 77843, USA
2. University of Hawaii Maui College, Department of Science, Technology, Engineering and Mathematics, Kahului, HI, 96732, USA
3. NOAA's Atlantic Oceanographic and Meteorological Laboratory, Miami, FL, 33149, USA

andreake@tamu.edu, katie.shamberger@tamu.edu, sdimarco@tamu.edu, kthyng@tamu.edu, hetland@tamu.edu, derek.manzello@noaa.gov, ian.enochs@noaa.gov, slowey@tamu.edu

*corresponding author: andreake@hawaii.edu

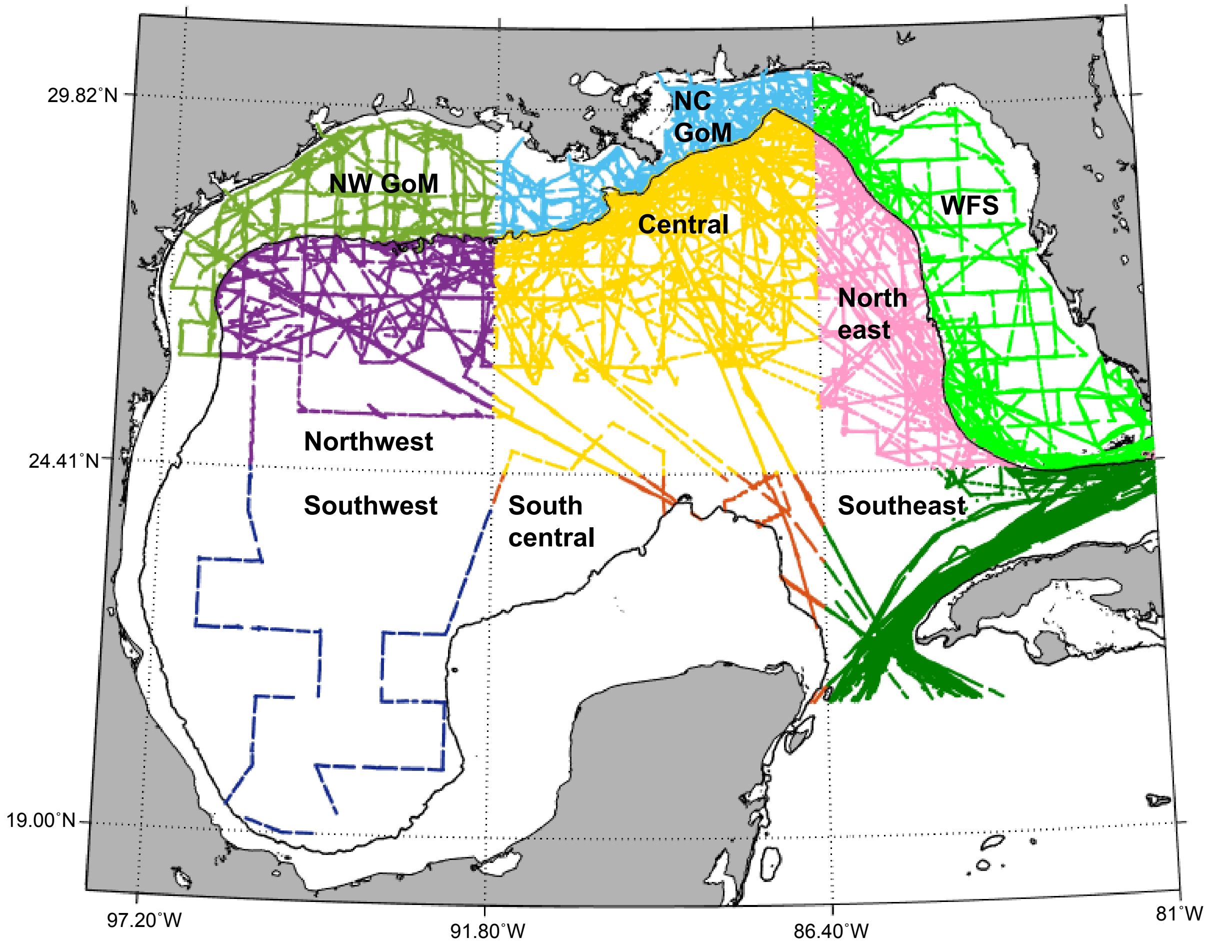

FIG S1: Open and coastal sub-regions for the long-term pCO_2 trend analysis. The coastal regions include the northwestern GoM (NW GoM), northcentral GoM (NC GoM) and West Florida Shelf (WFS). The open ocean regions include southwest, southcentral, southeast, northwest, central and northeast. The southwest and southcentral regions do not have enough data for the long-term trend analysis, and are not represented in the reported results. The solid black line represents the 200 m bathymetry line and separates the coastal and open ocean.

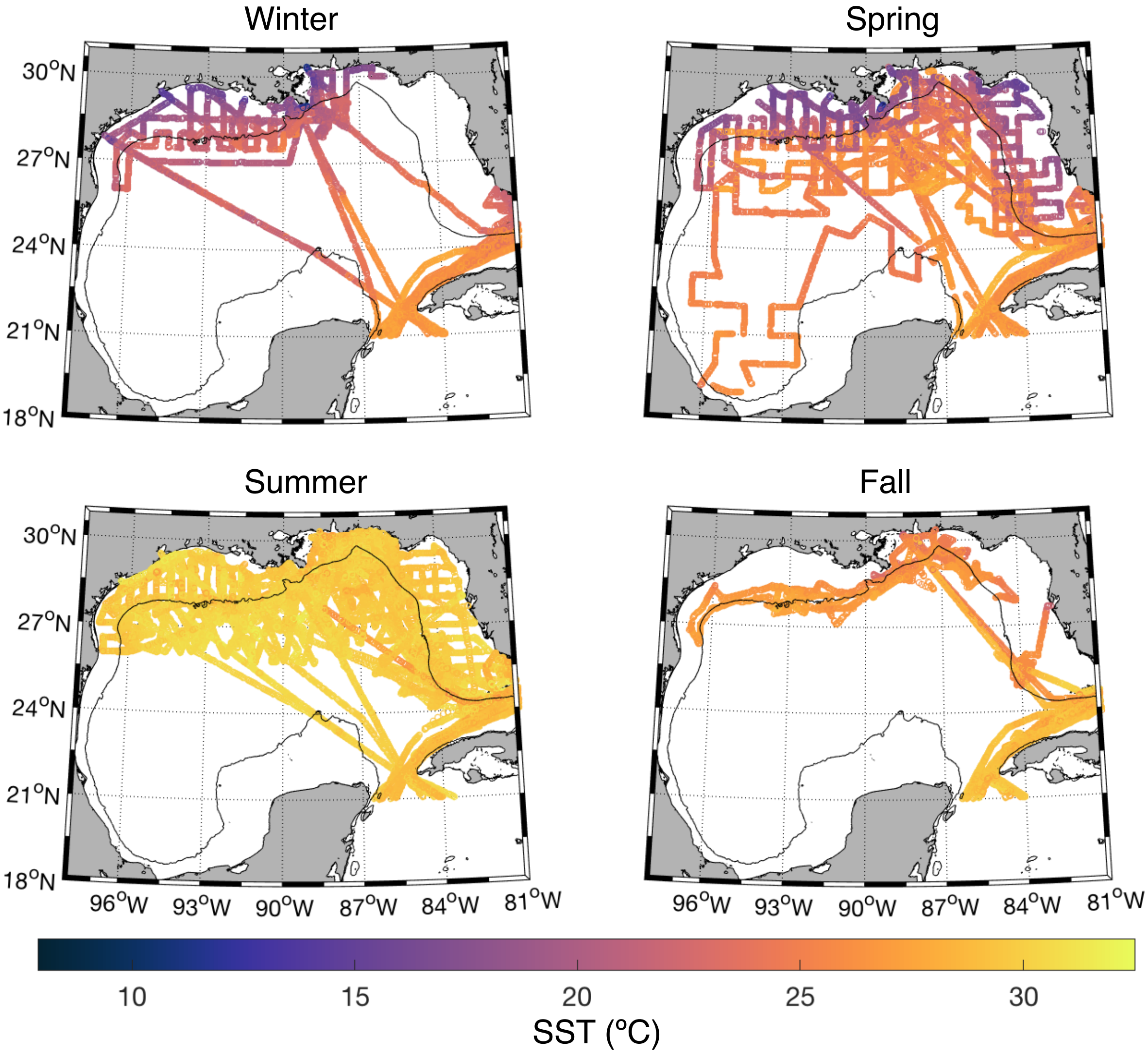

FIG S2: Seasonal surface seawater temperatures (SST) (°C). The solid black line represents the 200 m bathymetry contour.

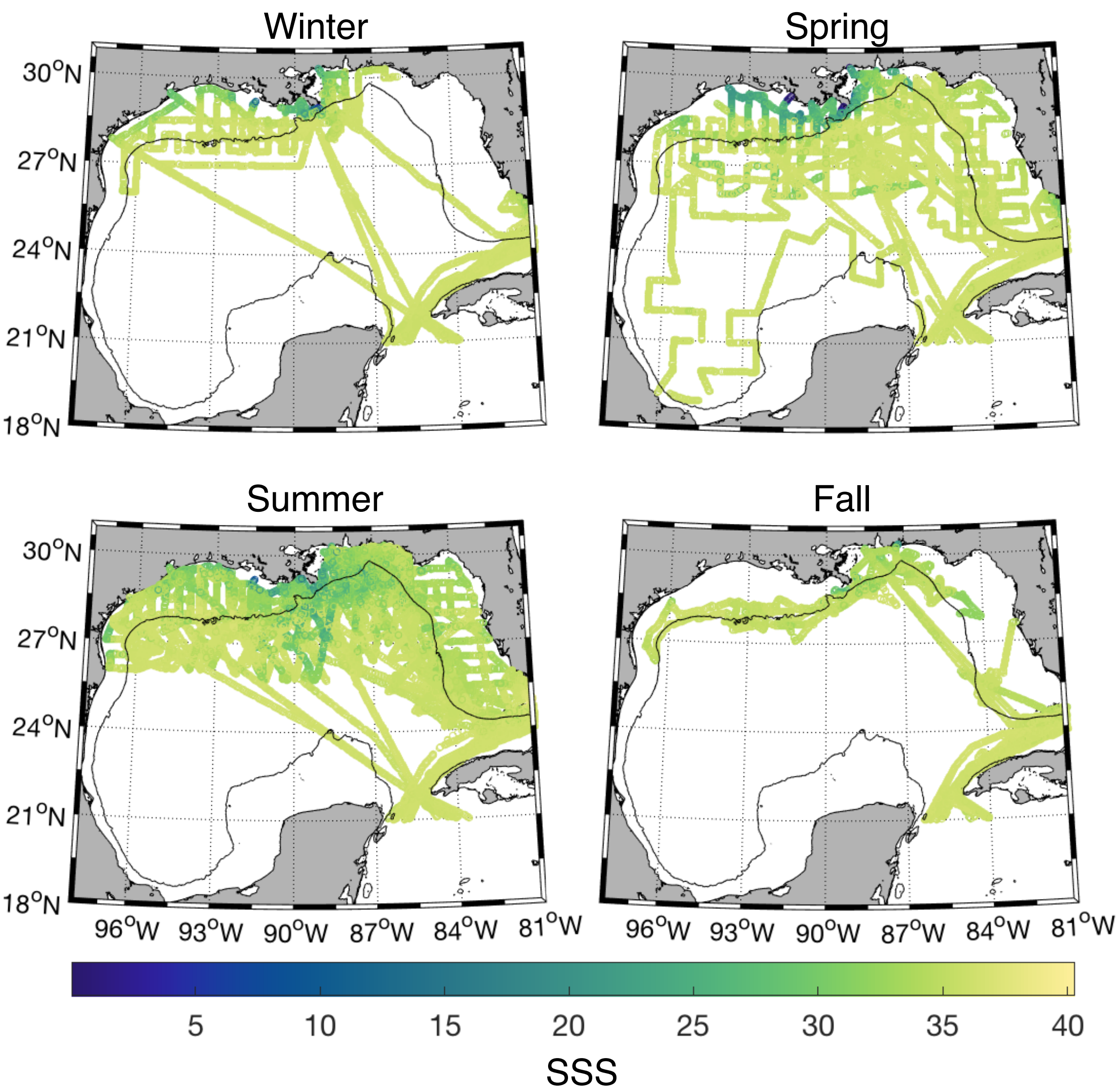

FIG S3: Seasonal sea surface salinity (SSS). The solid black line represents the 200 m bathymetry contour.

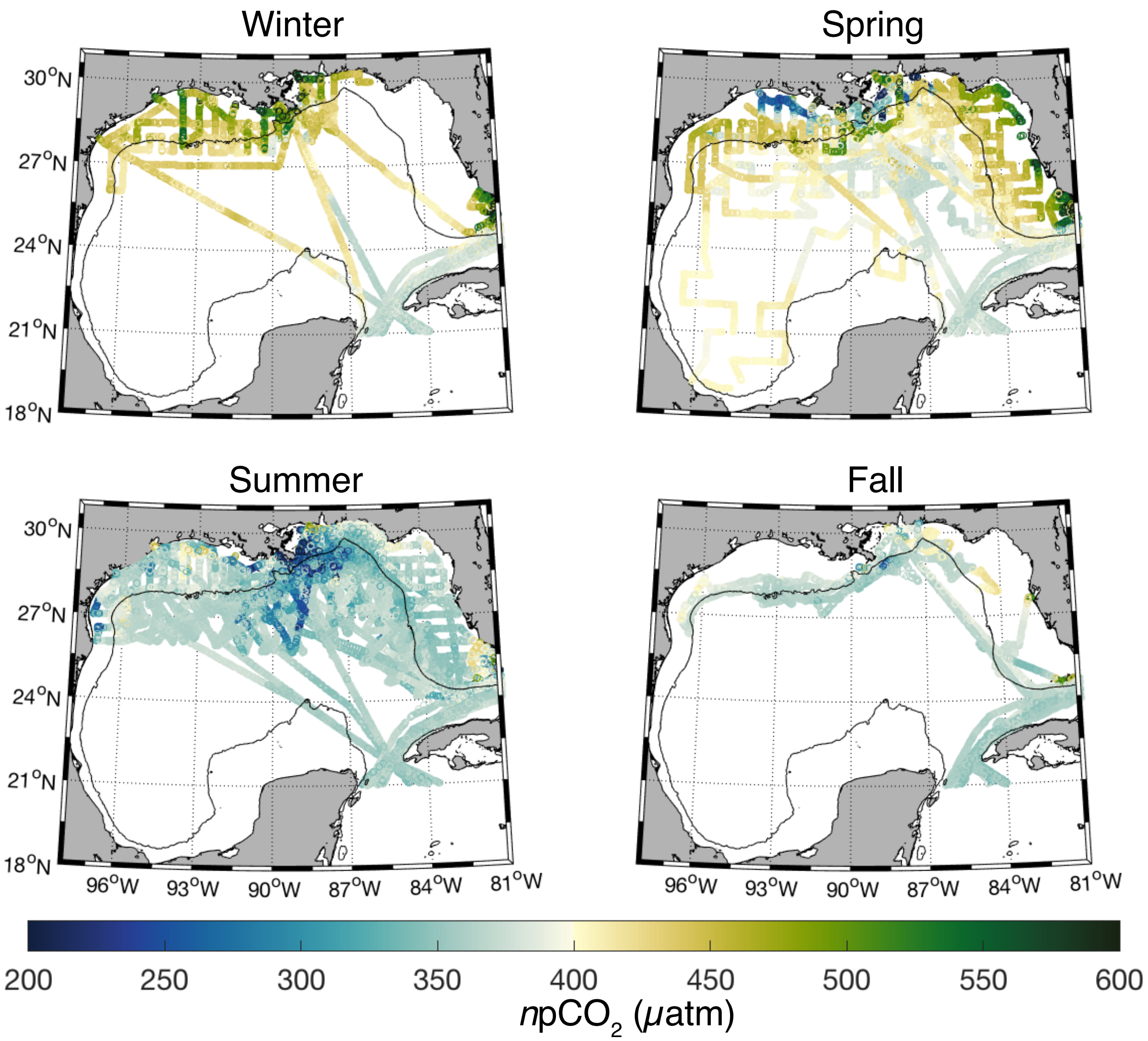

FIG S4: Temperature-normalized surface seawater pCO_2 ($npCO_2$, μatm) during winter, spring, summer and fall. Note that the actual minimum and maximum values are 66 and 3090 μatm, respectively. The solid black line represents the 200 m bathymetry contour.

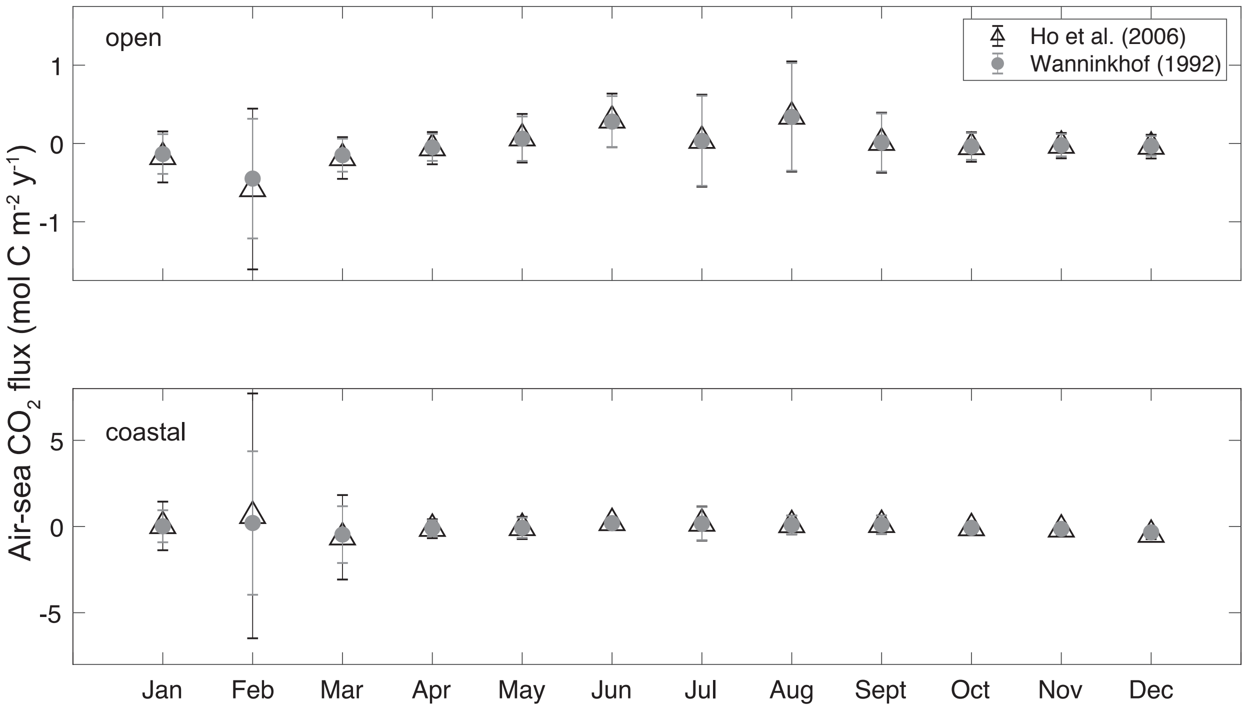

FIG S5: Monthly average (± std) air-sea CO_2 fluxes in the open (top panel) and coastal (bottom panel) ocean for the wind speed parameterization of Ho et al. (2006) (open triangles) and Wanninkhof et al. (1992) (closed circles). The anomalous summer 2009 (Jun-Sept) has been removed from the open ocean data.

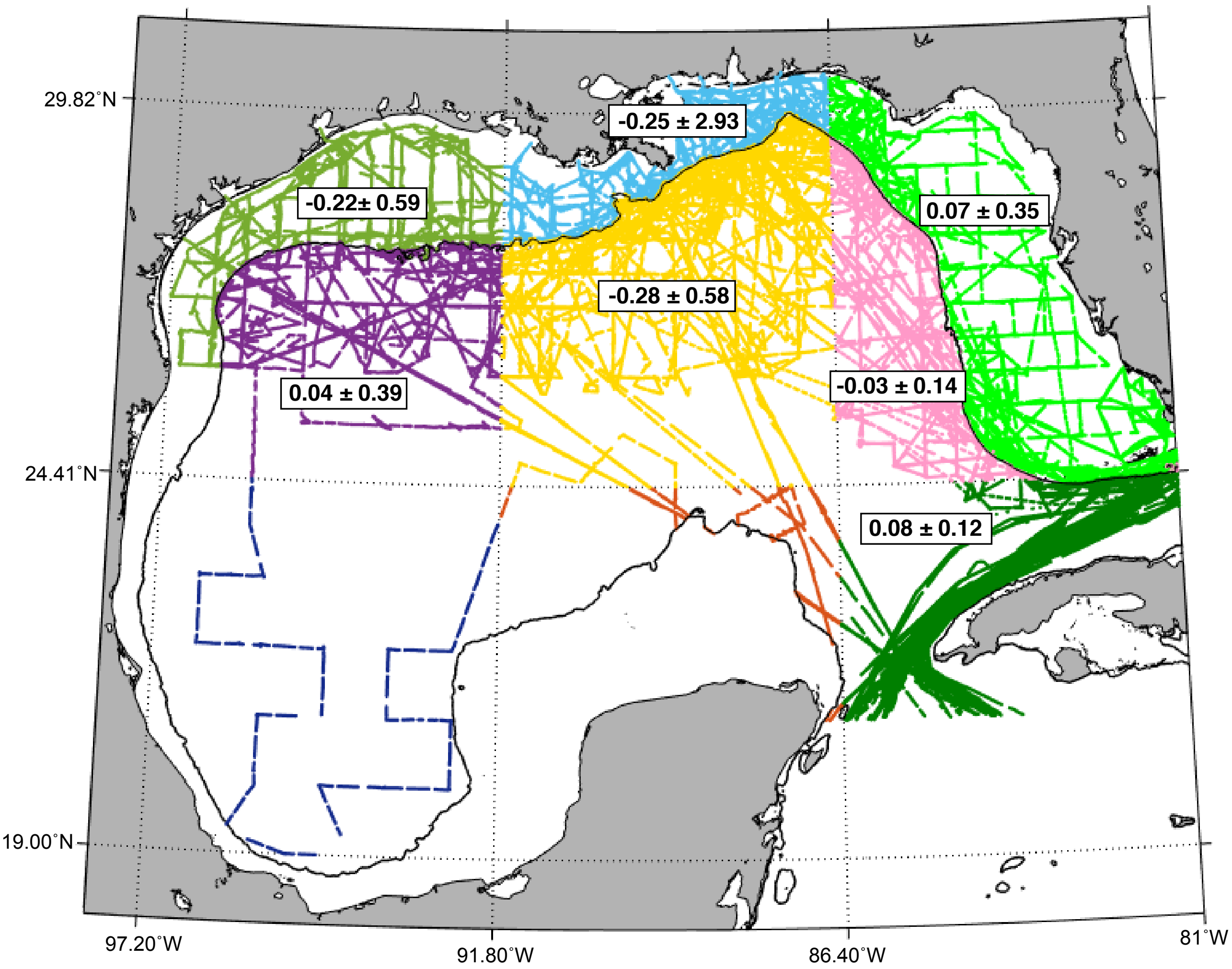

FIG S6: Annual CO_2 fluxes (mol C m^{-2} yr^{-1}) within each subregion of the GoM. The solid black line represents the 200 m bathymetry contour.

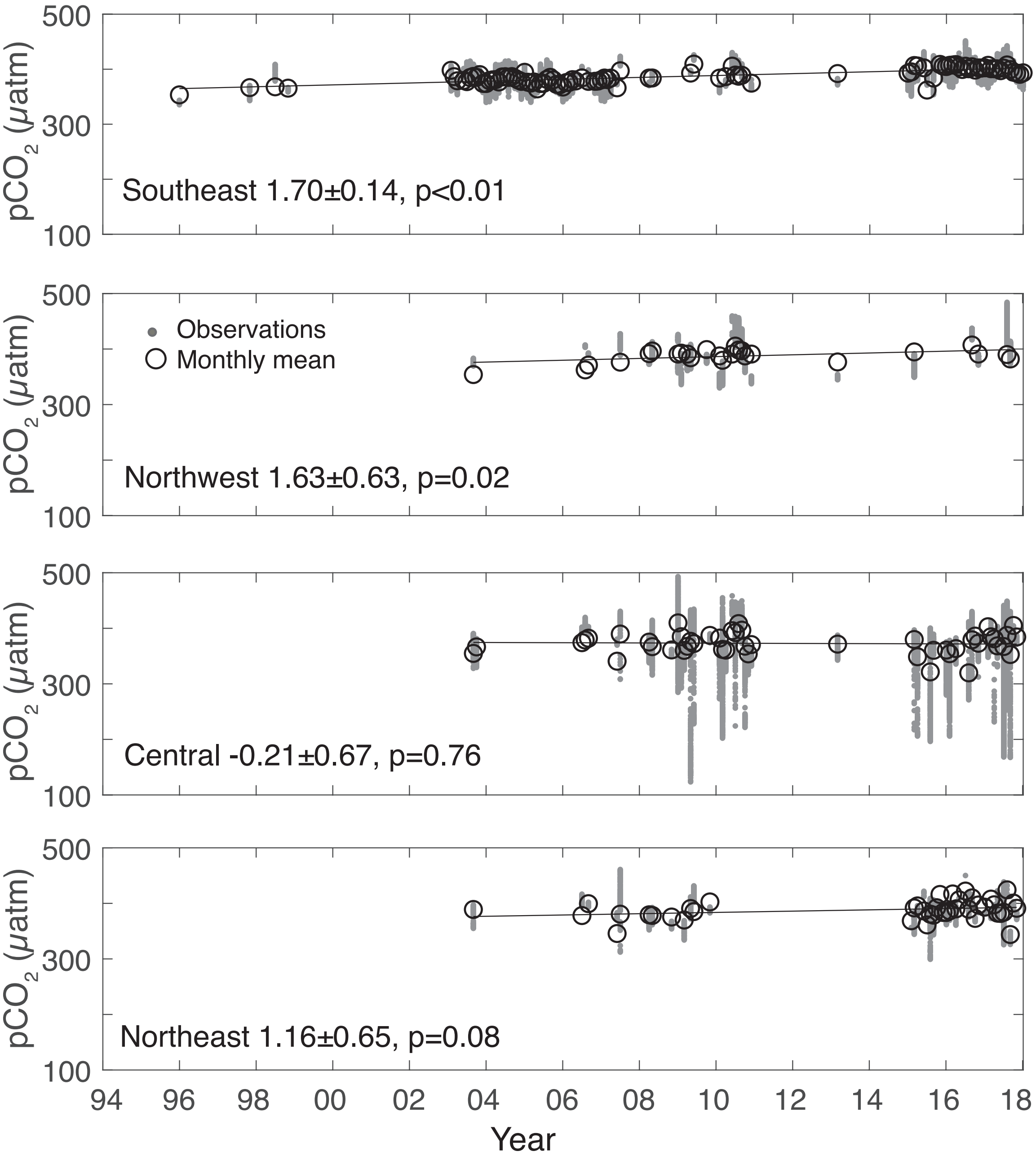

FIG S7: Long-term pCO_2 trends in open ocean sub-regions. The closed circles represent individual measurements and the open circles are monthly deseasonalized means.

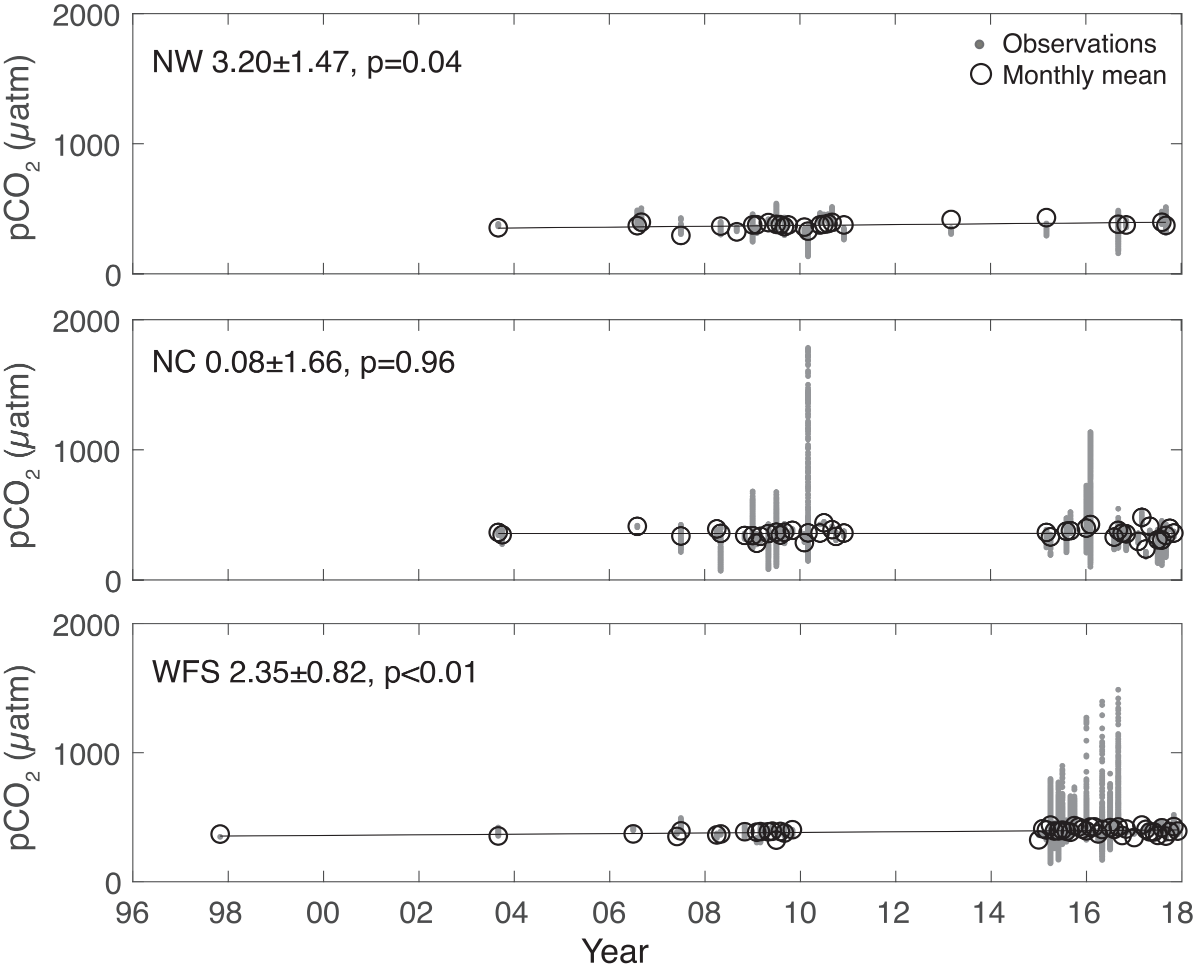

FIG S8: Long-term pCO_2 trends in coastal ocean sub-regions. The closed circles represent individual measurements and the open circles are monthly deseasonalized means.

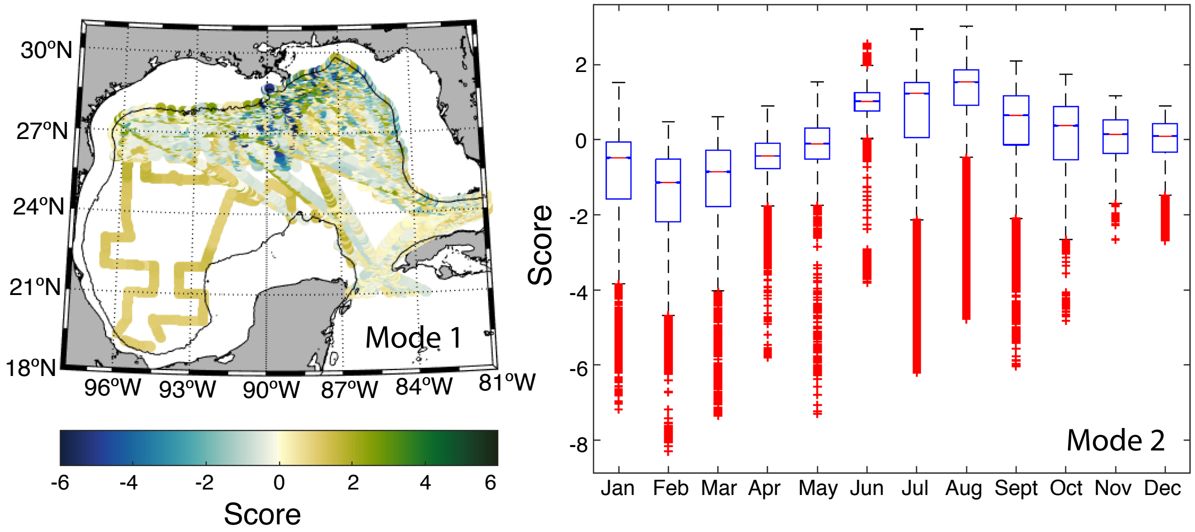

FIG S9: Open ocean spatial pattern showing scores of Mode 1 (left) and open ocean seasonal pattern showing scores of Mode 2 (right). The solid black line on the map (left) represents the 200 m bathymetry contour. In the box plot, the central red line is the median and the outer edges of the box represent the 25th and 75th percentiles. Whiskers extend to the most extreme points that are not considered outliers, and the outliers are plotted in red (+).

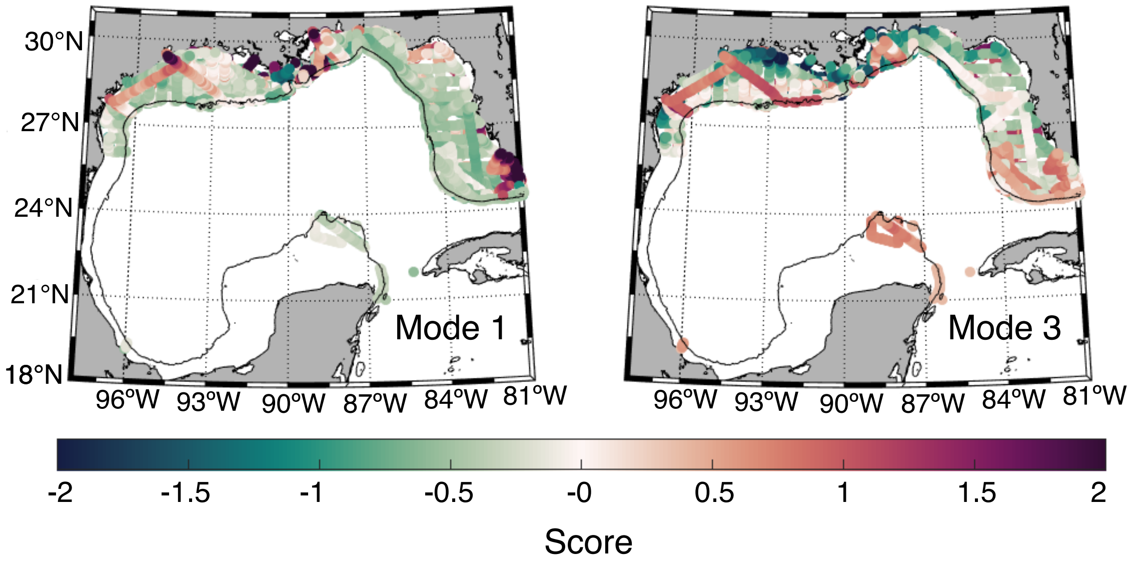

FIG S10: Coastal ocean spatial patterns in Mode 1 (left panel) and Mode 3 (right panel). The color bar represents the scores of the EOF. The solid black line on the maps represents the 200 m bathymetry contour.

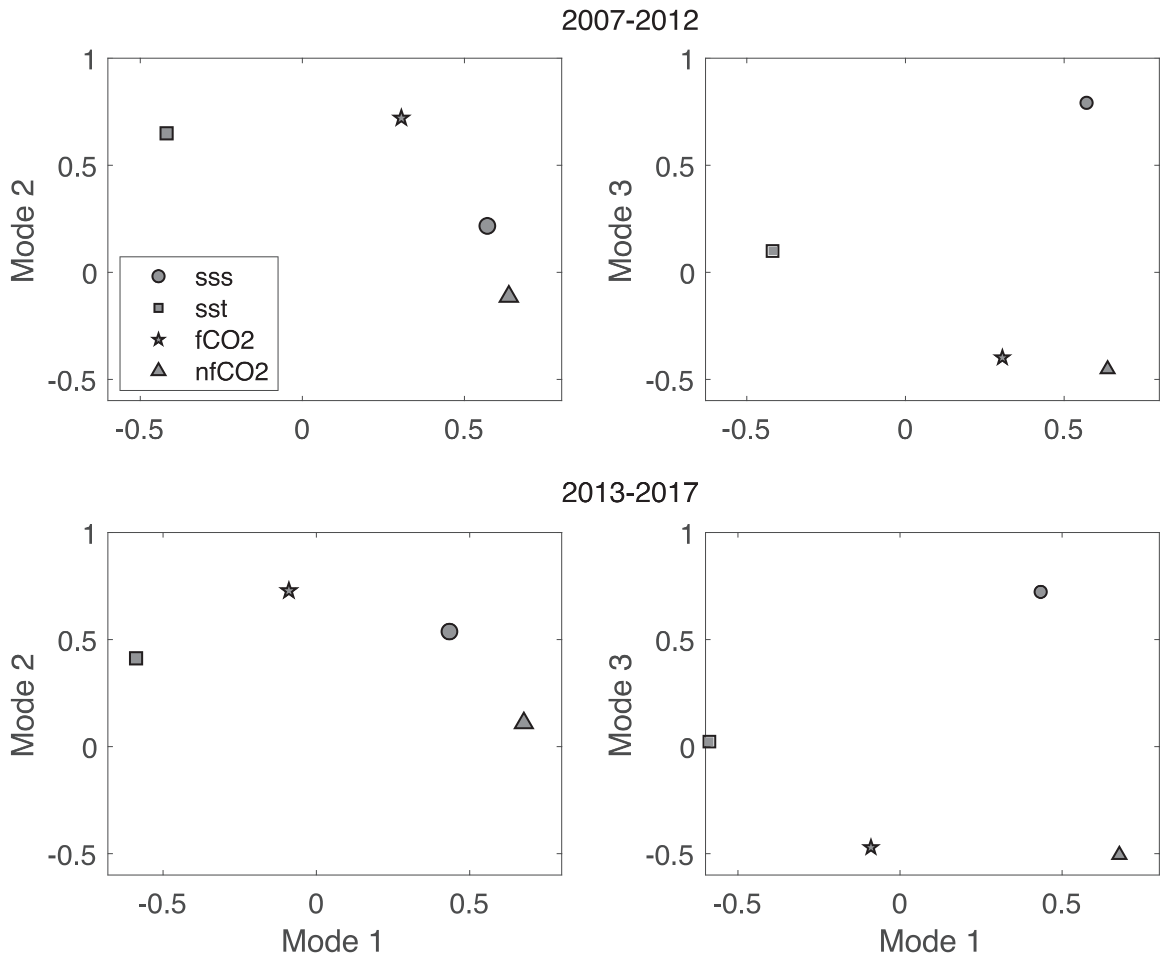

FIG S11: EOF analysis of open ocean modes for the years 2007-2012 (top panels) and 2013-2017 (bottom panels).

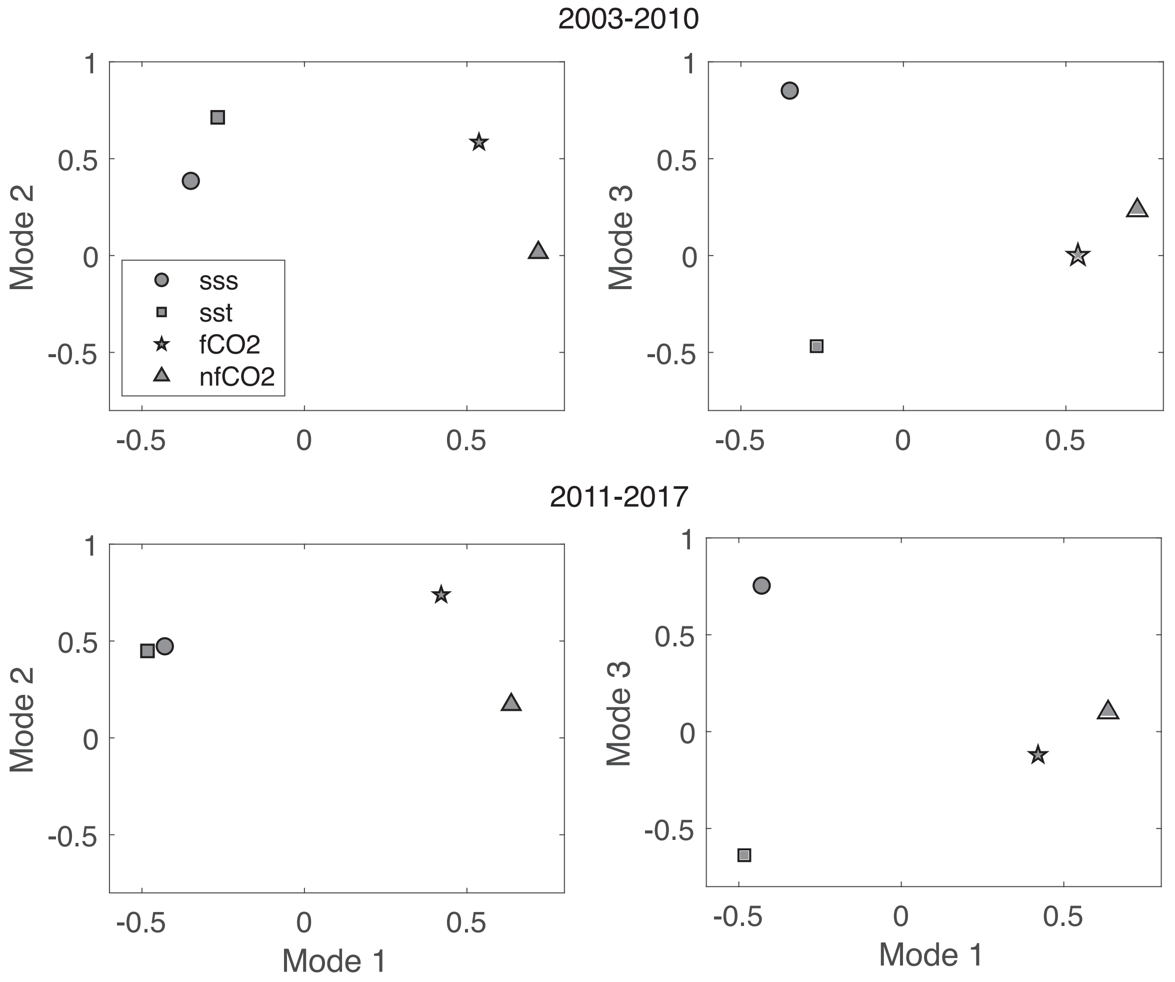

FIG S12: EOF analysis of coastal ocean modes for the years 2003-2010 (top panels) and 2011-2017 (bottom panels).

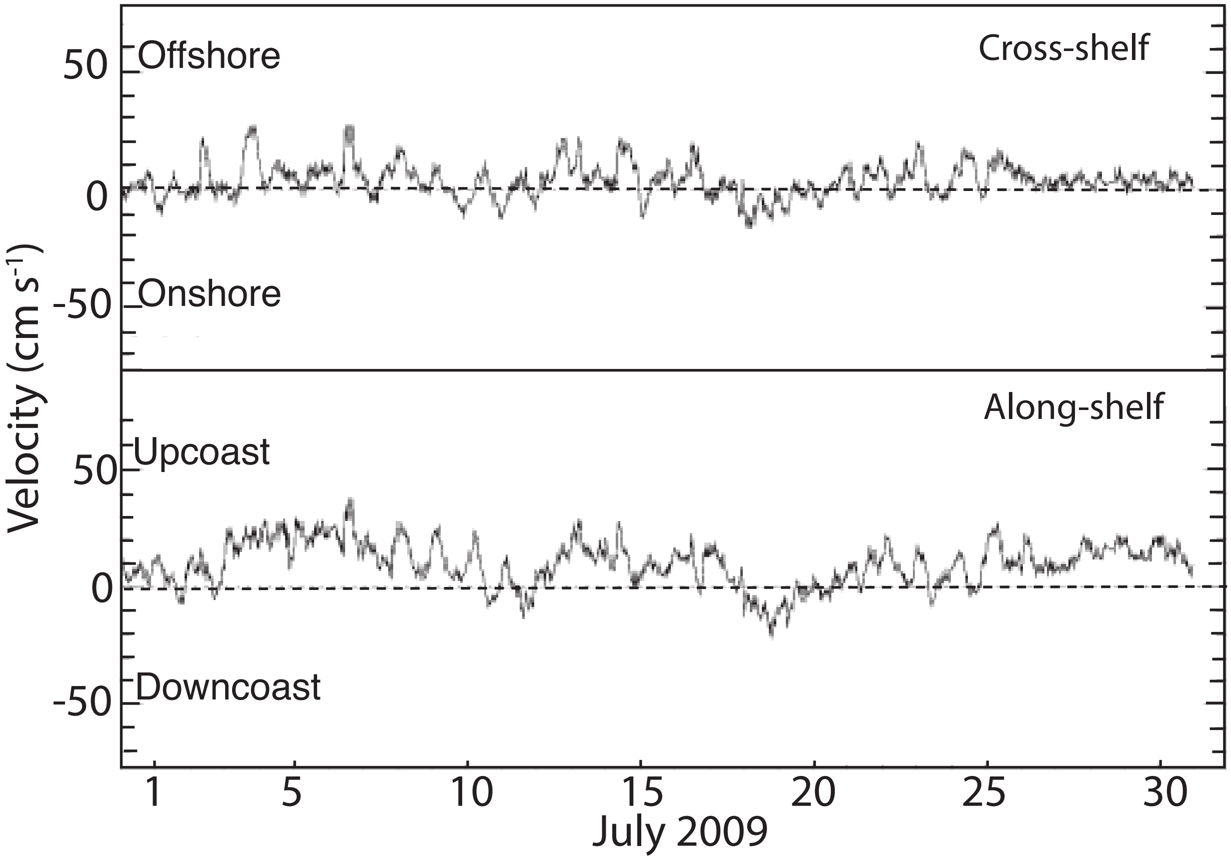

FIG S13: Cross-shelf (top panel) and along-shelf (bottom panel) currents collected at Texas Automated Buoy System (TABS) Buoy R located in the northern GoM (29° 38.100 N, 93° 38.502 W) during July 2009. Upcoast is eastward and downcoast is westward.

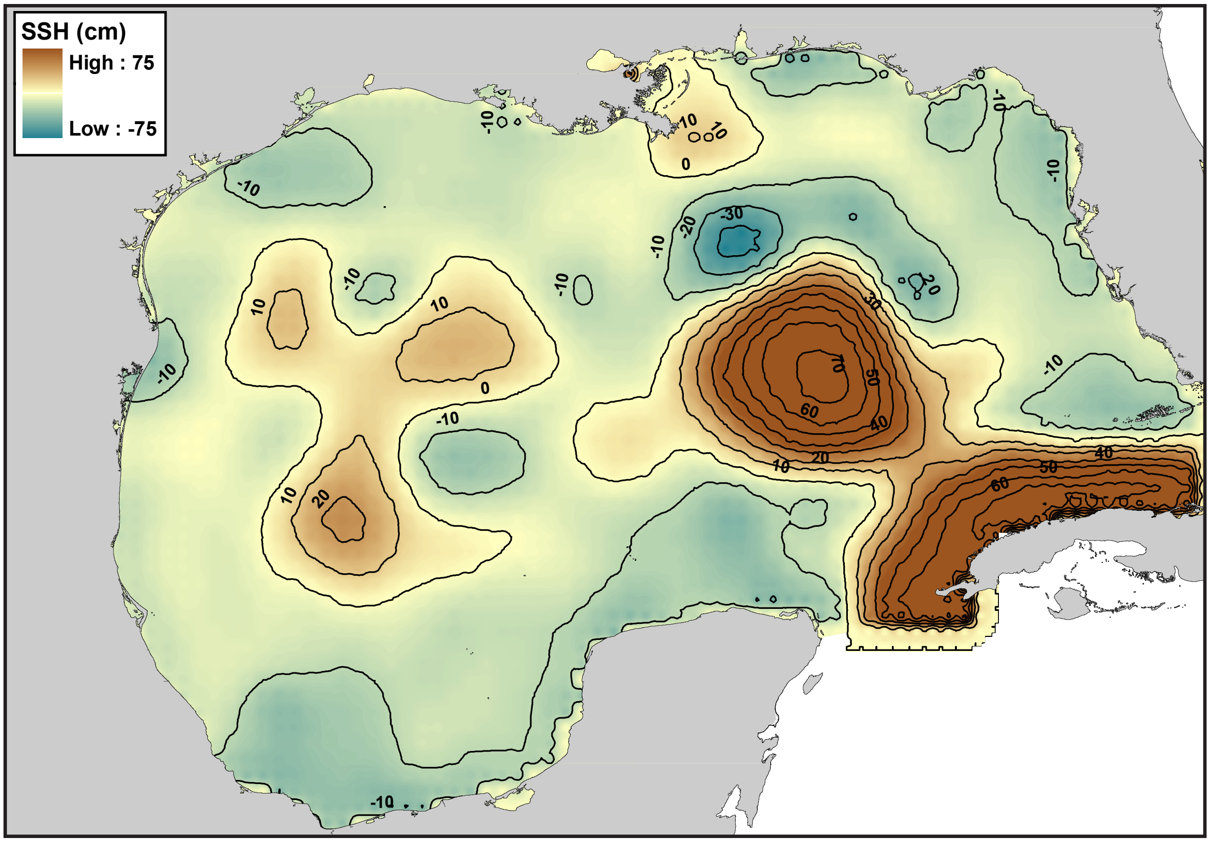

FIG S14: Sea surface height anomalies (cm) in the Gulf of Mexico on 07/20/2009. These data were obtained from the Colorado Center for Astrodynamics Research (CCAR). Note there are no data for Caribbean waters.

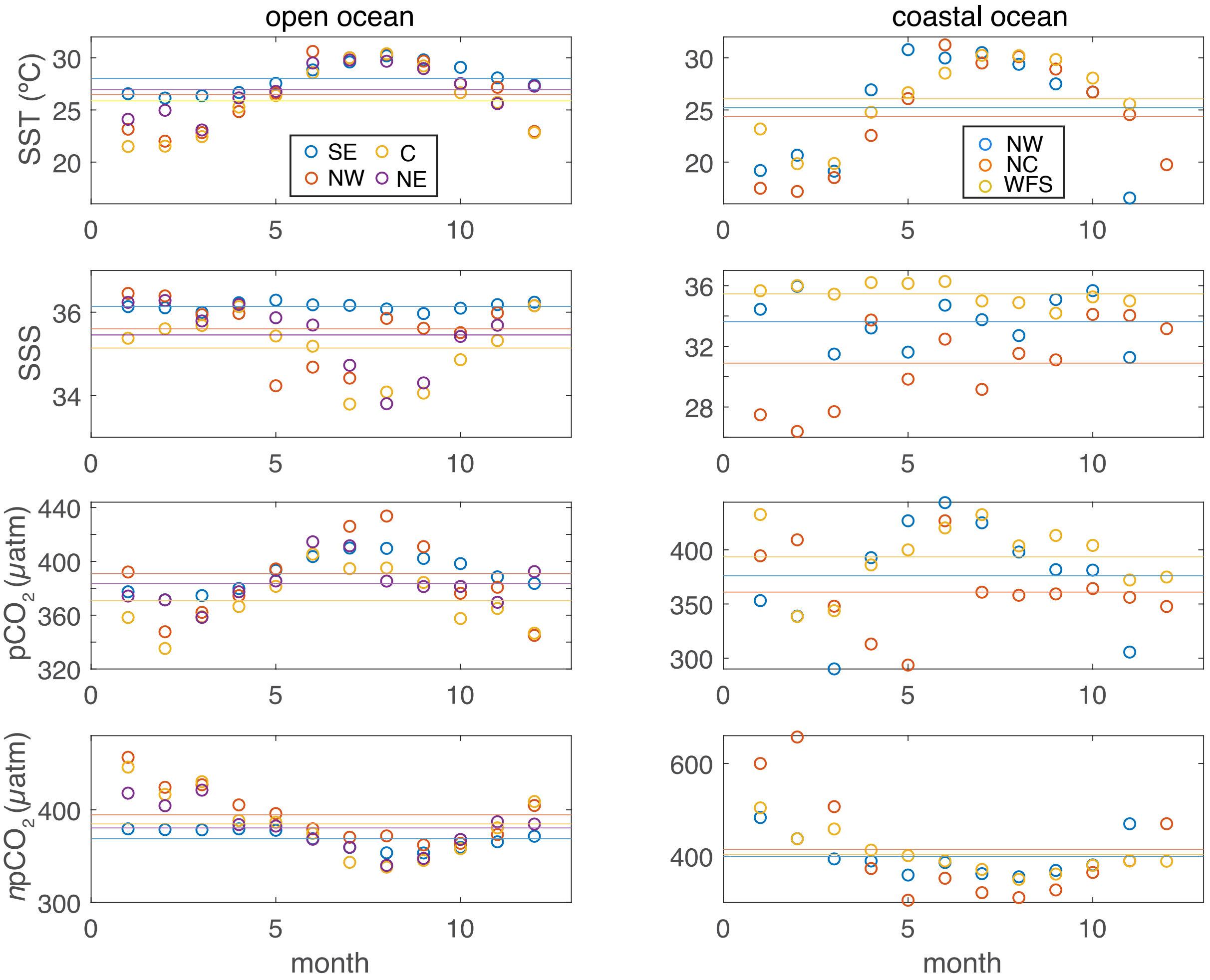

FIG S15: Seasonal trends (i.e., monthly means for entire dataset) for the open ocean bins (left panels; SE is southeast, NW is northwest, C is central and NE is northeast) and coastal ocean bins (right panels; NW is northwest, NC is northcentral and WFS is West Florida Shelf). The solid lines represent the annual mean, calculated from the monthly means.

Table S1: Number of measurements collected within each season and each region.

	Winter	Spring	Summer	Fall
Open	40460	75837	96591	29458
Coast	15643	40835	69231	12811
Total	56103	116672	165822	42269

Table S2: Statistical summary of long-term, deseasonalized pCO_2, $npCO_2$, SSS and SST trends by sub-region. Statistically significant ($p<0.05$) trends are in bold.

Region	Subregion	pCO_2 (µatm yr^{-1})± SE	p-value	R^2	n
Coast	NW GoM	3.20±1.47	**0.04**	0.17	26
	NC GoM	0.08±1.66	0.96	0.00	41
	WFS	2.35±0.82	**<<0.01**	0.15	49
Open	Southeast	1.70±0.14	**<<0.01**	0.59	99
	Northwest	1.63±0.63	**0.02**	0.23	25
	Central	-0.21±0.67	0.76	0.00	48
	Northeast	1.16±0.65	0.08	0.07	42
		***n*pCO2 (µatm yr^{-1})± SE**			
Coast	NW GoM	2.57±1.24	**0.05**	0.15	26
	NC GoM	1.04±3.13	0.74	0.00	41
	WFS	-0.09±0.90	0.93	0.00	49
Open	Southeast	1.19±0.12	**<<0.01**	0.50	98
	Northwest	0.87±0.48	0.08	0.12	25
	Central	-0.39±0.55	0.48	0.01	47
	Northeast	0.15±0.58	0.80	0.00	42
		**SSS± SE**			
Coast	NW GoM	0.06±0.11	0.61	0.01	26
	NC GoM	-0.29±0.14	**0.05**	0.10	38
	WFS	0.01±0.05	0.87	0.00	38
Open	Southeast	0.00±0.00	0.34	0.01	99
	Northwest	0.01±0.03	0.67	0.01	25
	Central	-0.08±0.04	0.07	0.08	48
	Northeast	-0.06±0.52	0.23	0.05	42
		**SST (°C)± SE**			
Coast	NW GoM	0.06±0.05	0.23	0.06	26
	NC GoM	-0.06±0.05	0.27	0.03	38
	WFS	0.07±0.04	0.06	0.09	39
Open	Southeast	0.03±0.01	**<<0.01**	0.14	99
	Northwest	0.03±0.04	0.39	0.03	25
	Central	0.01±0.04	0.83	0.00	48
	Northeast	0.06±0.04	0.16	0.05	42

Table S3: Time-series of observed variance for each open ocean mode for the years 2007-2012 and 2013-2017.

	Mode 1	Mode 2	Mode 3	Mode 4
2007-2012	56%	36%	8%	<1%
2013-2017	50%	44%	5%	<1%

Table S4: Time-series of observed variance for each coastal ocean mode for the years 2003-2010 and 2011-2017.

	Mode 1	Mode 2	Mode 3	Mode 4
2003-2010	46%	34%	20%	<1%
2011-2017	58%	26%	15%	<1%

Table S5: Cruise IDs and principle investigators for the SOCAT data used in this study.

CRUISE ID	Investigator/Observer
32KZ20090109	Cai, W.-J.
32KZ20090719	Cai, W.-J.
32KZ20100311	Cai, W.-J.
33GG20080403	Wanninkhof, R.
33GG20080419	Wanninkhof, R.
33GG20080512	Wanninkhof, R.
33GG20080920	Wanninkhof, R.
33GG20081114	Wanninkhof, R.
33GG20090204	Wanninkhof, R.
33GG20090227	Wanninkhof, R.
33GG20090407	Wanninkhof, R.
33GG20090419	Wanninkhof, R.
33GG20090513	Wanninkhof, R.
33GG20090611	Wanninkhof, R.
33GG20090701	Wanninkhof, R.
33GG20090727	Wanninkhof, R.
33GG20090830	Wanninkhof, R.
33GG20090915	Wanninkhof, R.
33GG20091019	Wanninkhof, R.
33GG20091101	Wanninkhof, R.
33GG20150303	Wanninkhof, R. : Pierrot, D.
33GG20150317	Wanninkhof, R. : Pierrot, D.
33GG20150730	Wanninkhof, R. : Pierrot, D.
33GG20150822	Wanninkhof, R. : Pierrot, D.
33GG20150912	Wanninkhof, R. : Pierrot, D.

33GG20160410	Wanninkhof, R. : Pierrot, D.
33GG20160809	Wanninkhof, R. : Pierrot, D.
33GG20160902	Wanninkhof, R. : Pierrot, D.
33GG20160918	Wanninkhof, R. : Pierrot, D.
33GG20161025	Wanninkhof, R. : Pierrot, D.
33GG20161103	Wanninkhof, R. : Pierrot, D.
33GG20161118	Wanninkhof, R. : Pierrot, D.
33GG20170505	Wanninkhof, R. : Pierrot, D.
33GG20170702	Wanninkhof, R. : Pierrot, D.
33GG20170721	Wanninkhof, R. : Pierrot, D.
33GG20170809	Wanninkhof, R. : Pierrot, D.
33GG20170904	Wanninkhof, R. : Pierrot, D.
33GG20170917	Wanninkhof, R. : Pierrot, D.
33GG20171010	Wanninkhof, R. : Pierrot, D.
33GG20171112	Wanninkhof, R. : Pierrot, D.
33H320130228	Takahashi, T. : Sweeney, C. : Sutherland, S.C.
33KF20030215	Wanninkhof, R.
33KF20030302	Wanninkhof, R.
33KF20030316	Wanninkhof, R.
33KF20030330	Wanninkhof, R.
33KF20030412	Wanninkhof, R.
33KF20030621	Wanninkhof, R.
33KF20030706	Wanninkhof, R.
33KF20030720	Wanninkhof, R.
33KF20030802	Wanninkhof, R.
33KF20030816	Wanninkhof, R.
33KF20030831	Wanninkhof, R.
33KF20030913	Wanninkhof, R.
33KF20030927	Wanninkhof, R.
33KF20031011	Wanninkhof, R.
33KF20031109	Wanninkhof, R.
33KF20031123	Wanninkhof, R.
33KF20031221	Wanninkhof, R.
33KF20040104	Wanninkhof, R.
33KF20040118	Wanninkhof, R.
33KF20040201	Wanninkhof, R.
33KF20040303	Wanninkhof, R.
33KF20040314	Wanninkhof, R.
33KF20040328	Wanninkhof, R.

33KF20040410	Wanninkhof, R.
33KF20040424	Wanninkhof, R.
33KF20040512	Wanninkhof, R.
33KF20040523	Wanninkhof, R.
33KF20040607	Wanninkhof, R.
33KF20040620	Wanninkhof, R.
33KF20040705	Wanninkhof, R.
33KF20040718	Wanninkhof, R.
33KF20040801	Wanninkhof, R.
33KF20040815	Wanninkhof, R.
33KF20040830	Wanninkhof, R.
33KF20040907	Wanninkhof, R.
33KF20040912	Wanninkhof, R.
33KF20041010	Wanninkhof, R.
33KF20041024	Wanninkhof, R.
33KF20041107	Wanninkhof, R.
33KF20041122	Wanninkhof, R.
33KF20041206	Wanninkhof, R.
33KF20041220	Wanninkhof, R.
33KF20050103	Wanninkhof, R.
33KF20050117	Wanninkhof, R.
33KF20050214	Wanninkhof, R.
33KF20050228	Wanninkhof, R.
33KF20050314	Wanninkhof, R.
33KF20050328	Wanninkhof, R.
33KF20050410	Wanninkhof, R.
33KF20050424	Wanninkhof, R.
33KF20050508	Wanninkhof, R.
33KF20050605	Wanninkhof, R.
33KF20050620	Wanninkhof, R.
33KF20050703	Wanninkhof, R.
33KF20050731	Wanninkhof, R.
33KF20050815	Wanninkhof, R.
33KF20050828	Wanninkhof, R.
33KF20050912	Wanninkhof, R.
33KF20051009	Wanninkhof, R.
33KF20051107	Wanninkhof, R.
33KF20051120	Wanninkhof, R.
33KF20051205	Wanninkhof, R.

33KF20060116	Wanninkhof, R.
33KF20060129	Wanninkhof, R.
33KF20060213	Wanninkhof, R.
33KF20060227	Wanninkhof, R.
33KF20060313	Wanninkhof, R.
33KF20060327	Wanninkhof, R.
33KF20060409	Wanninkhof, R.
33KF20060423	Wanninkhof, R.
33KF20060508	Wanninkhof, R.
33KF20061120	Wanninkhof, R.
33KF20061204	Wanninkhof, R.
33KF20061218	Wanninkhof, R.
33KF20070101	Wanninkhof, R.
33KF20070115	Wanninkhof, R.
33KF20070129	Wanninkhof, R.
33KF20070211	Wanninkhof, R.
33KF20070226	Wanninkhof, R.
33KF20070311	Wanninkhof, R.
33KF20070325	Wanninkhof, R.
33KF20070408	Wanninkhof, R.
33KF20070422	Wanninkhof, R.
33KF20150104	Wanninkhof, R. : Pierrot, D.
33KF20150118	Wanninkhof, R. : Pierrot, D.
33KF20150124	Wanninkhof, R. : Pierrot, D.
33KF20150207	Wanninkhof, R. : Pierrot, D.
33MW19960120	Wanninkhof, R.
33RO19971111	Wanninkhof, R.
33RO19980712	Wanninkhof, R.
33RO19981117	Wanninkhof, R.
33RO20030828	Wanninkhof, R.
33RO20030909	Wanninkhof, R.
33RO20030921	Wanninkhof, R.
33RO20031006	Wanninkhof, R.
33RO20060727	Wanninkhof, R.
33RO20060822	Wanninkhof, R.
33RO20060914	Wanninkhof, R.
33RO20070604	Wanninkhof, R.
33RO20070711	Wanninkhof, R.
33RO20090819	Wanninkhof, R.

33RO20090914	Wanninkhof, R.
33RO20101015	Wanninkhof, R.
33WA20150413	Wanninkhof, R.
33WA20150422	Wanninkhof, R. : Pierrot, D.
33WA20150507	Millero, F. : Wanninkhof, R.
33WA20150601	Millero, F. : Wanninkhof, R.
33WA20150610	Millero, F. : Wanninkhof, R.
33WA20150727	Millero, F. : Wanninkhof, R.
33WA20150822	Millero, F. : Wanninkhof, R.
33WA20150928	Millero, F. : Wanninkhof, R.
33WA20160104	Millero, F. : Wanninkhof, R.
33WA20160115	Millero, F. : Wanninkhof, R.
33WA20160509	Millero, F. : Wanninkhof, R.
33WA20160725	Millero, F. : Wanninkhof, R.
33WA20160919	Millero, F. : Wanninkhof, R.
33WA20170130	Millero, F. : Wanninkhof, R.
33WA20170515	Millero, F. : Wanninkhof, R.
74X120100130	Millero, F. : Wanninkhof, R.
74X120100419	Millero, F. : Wanninkhof, R.
74X120100616	Wanninkhof, R.
74X120100707	Wanninkhof, R.
74X120100801	Wanninkhof, R.
74X120100826	Wanninkhof, R.
74X120100915	Wanninkhof, R.
74X120101214	Wanninkhof, R.
BHAF20160417	Wanninkhof, R. : Pierrot, D.
BHAF20160501	Wanninkhof, R. : Pierrot, D.
BHAF20160515	Wanninkhof, R. : Pierrot, D.
BHAF20160529	Wanninkhof, R. : Pierrot, D.
BHAF20160619	Wanninkhof, R. : Pierrot, D.
BHAF20160710	Wanninkhof, R. : Pierrot, D.
BHAF20160731	Wanninkhof, R. : Pierrot, D.
BHAF20160925	Wanninkhof, R. : Pierrot, D.
BHAF20161002	Wanninkhof, R. : Pierrot, D.
BHAF20161016	Wanninkhof, R. : Pierrot, D.
BHAF20161030	Wanninkhof, R. : Pierrot, D.
BHAF20161113	Wanninkhof, R. : Pierrot, D.
BHAF20161127	Wanninkhof, R. : Pierrot, D.
BHAF20161211	Wanninkhof, R. : Pierrot, D.

BHAF20161218	Wanninkhof, R. : Pierrot, D.
BHAF20170102	Wanninkhof, R. : Pierrot, D.
BHAF20170122	Wanninkhof, R. : Pierrot, D.
BHAF20170205	Wanninkhof, R. : Pierrot, D.
BHAF20170305	Wanninkhof, R. : Pierrot, D.
BHAF20170319	Wanninkhof, R. : Pierrot, D.
BHAF20170402	Wanninkhof, R. : Pierrot, D.
BHAF20170416	Wanninkhof, R. : Pierrot, D.
BHAF20170430	Wanninkhof, R. : Pierrot, D.
BHAF20170521	Wanninkhof, R. : Pierrot, D.
BHAF20170611	Wanninkhof, R. : Pierrot, D.
BHAF20170813	Wanninkhof, R. : Pierrot, D.
BHAF20170903	Wanninkhof, R. : Pierrot, D.
BHAF20170917	Wanninkhof, R. : Pierrot, D.
BHAF20170924	Wanninkhof, R. : Pierrot, D.
BHAF20171001	Wanninkhof, R. : Pierrot, D.
BHAF20171008	Wanninkhof, R. : Pierrot, D.
BHAF20171015	Wanninkhof, R. : Pierrot, D.
BHAF20171022	Wanninkhof, R. : Pierrot, D.
BHAF20171029	Wanninkhof, R. : Pierrot, D.
BHAF20171105	Wanninkhof, R. : Pierrot, D.
BHAF20171203	Wanninkhof, R. : Pierrot, D.
BHAF20171231	Wanninkhof, R. : Pierrot, D.
MLCE20150223	Wanninkhof, R. : Pierrot, D.
MLCE20150316	Wanninkhof, R. : Pierrot, D.
MLCE20150406	Wanninkhof, R. : Pierrot, D.
MLCE20151109	Wanninkhof, R. : Pierrot, D.
MLCE20151130	Wanninkhof, R. : Pierrot, D.
MLCE20151221	Wanninkhof, R. : Pierrot, D.
MLCE20160111	Wanninkhof, R. : Pierrot, D.
MLCE20160201	Wanninkhof, R. : Pierrot, D.
MLCE20160222	Wanninkhof, R. : Pierrot, D.
MLCE20160314	Wanninkhof, R. : Pierrot, D.
MLCE20160404	Wanninkhof, R. : Pierrot, D.
MLCE20161107	Wanninkhof, R. : Pierrot, D.
MLCE20161128	Wanninkhof, R. : Pierrot, D.
MLCE20170102	Wanninkhof, R. : Pierrot, D.
MLCE20170109	Wanninkhof, R. : Pierrot, D.
MLCE20170130	Wanninkhof, R. : Pierrot, D.

MLCE20170220	Wanninkhof, R. : Pierrot, D.
MLCE20170313	Wanninkhof, R. : Pierrot, D.
MLCE20170403	Wanninkhof, R. : Pierrot, D.
MLCE20170424	Wanninkhof, R. : Pierrot, D.
MLCE20170505	Wanninkhof, R. : Pierrot, D.
MLCE20170526	Wanninkhof, R. : Pierrot, D.
MLCE20170604	Wanninkhof, R. : Pierrot, D.
MLCE20170618	Wanninkhof, R. : Pierrot, D.
MLCE20170702	Wanninkhof, R. : Pierrot, D.
MLCE20170716	Wanninkhof, R. : Pierrot, D.
MLCE20170730	Wanninkhof, R. : Pierrot, D.
MLCE20170813	Wanninkhof, R. : Pierrot, D.
MLCE20170915	Wanninkhof, R. : Pierrot, D.
MLCE20171006	Wanninkhof, R. : Pierrot, D.
MLCE20171027	Wanninkhof, R. : Pierrot, D.
MLCE20171106	Wanninkhof, R. : Pierrot, D.
MLCE20171116	Wanninkhof, R. : Pierrot, D.
MLCE20171202	Wanninkhof, R. : Pierrot, D.
MLCE20171216	Wanninkhof, R. : Pierrot, D.
MLCE20171230	Wanninkhof, R. : Pierrot, D.
